# Stability and integrity of self-assembled bovine parvovirus virus‑like particles (BPV‑VLPs) of VP2 and combination of VP1VP2 assisted by baculovirus-insect cell expression: a potential logistical platform for vaccine deployment

**DOI:** 10.1186/s12985-024-02322-0

**Published:** 2024-04-19

**Authors:** Ashenafi Kiros Wubshet, Guo-xiu Li, Qian Li, Jun-Fei Dai, Yao-Zhong Ding, Luoyi Zhou, Min Qu, Yang Wang, Zhongyuan Ma, Gebremeskel Mamu Werid, Birhanu Hadush Abera, Asmelash Tassew Kebede, Yuefeng Sun, Xiangping Yin, Yongsheng Liu, Zhang Jie

**Affiliations:** 1 State Key Laboratory of Veterinary Etiological Biology, National/OIE Foot and Mouth Disease Reference Laboratory, Lanzhou Veterinary Research Institute, Chinese Academy of Agricultural Sciences, Lanzhou, 730046 People’s Republic of China; 2 College of Animal Science & Technology (CAST), Hebei Normal University of Science & Technology (HNUST), Qinhuangdao, People’s Republic of China; 3 Department of Veterinary Basics and Diagnostic Sciences, College of Veterinary Science, Mekelle University, 2084 Mekelle, Tigray Ethiopia; 4 Department of Animal Science, College of Agriculture and Natural Resources, Raya University, 92, Maychew, Tigray Ethiopia; 5 Davies Livestock Research Centre, School of Animal & Veterinary Sciences, University of Adelaide, Roseworthy Campus, Roseworthy, SA 5371 Australia

**Keywords:** Bovine parvovirus, Physicochemical properties, Secondary structure, Virus-like particle, VP2, VP1VP2

## Abstract

**Background:**

Bovine parvovirus (BPV) is an autonomous DNA virus with a smaller molecular size and subtle differences in its structural proteins, unlike other animal parvoviruses. More importantly, this virus has the potential to produce visible to silent economic catastrophes in the livestock business, despite receiving very little attention. Parvoviral virus-like particles (VLPs) as vaccines and as logistical platforms for vaccine deployment are well studied. However, no single experimental report on the role of VP1 in the assembly and stability of BPV-VLPs is available. Furthermore, the self-assembly, integrity and stability of the VLPs of recombinant BPV VP2 in comparison to VP1 VP2 Cap proteins using any expression method has not been studied previously. In this study, we experimentally evaluated the self-assembling ability with which BPV virus-like particles (VLPs) could be synthesized from a single structural protein (VP2) and by integrating both VP2 and VP1 amino acid sequences.

**Methods:**

*In silico* and experimental cloning methods were carried out. His-tagged and without-His-tag VP2 and V1VP2-encoding amino acid sequences were cloned and inserted into pFastbacdual, and insect cell-generated recombinant protein was evaluated by SDS‒PAGE and western blot. Period of infectivity and expression level were determined by IFA. The integrity and stability of the BPV VLPs were evaluated by transmission electron microscopy. The secondary structure of the BPV VLPs from both VP2 and V1VP2 was analyzed by circular dichroism.

**Results:**

Our findings show that VP2 alone was equally expressed and purified into detectable proteins, and the stability at different temperatures and pH values was not appreciably different between the two kinds of VLPs. Furthermore, BPV-VP2 VLPs were praised for their greater purity and integrity than BPV-VP1VP2 VLPs, as indicated by SDS‒PAGE. Therefore, our research demonstrates that the function of VP1 has no bearing on the stability or integrity of BPV-VLPs.

**Conclusions:**

In summary, incredible physiochemically stable BPV VP2-derived VLPs have been found to be promising candidates for the development of multivalent vaccines and immunodiagnostic kits against enteric viruses and to carry heterogeneous epitopes for various economically important livestock diseases.

**Supplementary Information:**

The online version contains supplementary material available at 10.1186/s12985-024-02322-0.

## Back ground

Parvoviruses are a group of very broad species-specific viruses that have been detected in cattle, pigs, dogs, cats, mink, geese, rats, mice, and humans. Abinati and Warfield identified BPV for the first time in the feces of calves in Maryland (USA) in 1961 [1]. Bovine parvovirus (BPV) [41] was classified as an autonomous parvovirus in 1970 and placed in the Parvoviridae family's bocaparvovirus genus [4, 6, 41]. This virus group is very similar to other autonomous parvoviruses in terms of its viral properties, with the exception of subtle differences in its structural proteins. Furthermore, Bates and coworkers [6] found little resemblance between bovine parvovirus and other animal parvoviruses [36]. More importantly, such virus groups have the potential to produce visible-to-silent economic catastrophes in the livestock business. The prevalence and genetic variability of BPV across diverse ecosystems and geographical settings are rarely studied, despite the year of its announcement in 1960 as a veterinary important virus. It has coinfection potential with bacteria and nonparvoviruses, causing respiratory and gastrointestinal disease and challenging the cattle industry worldwide. However, only a few pieces of information and reports are available, with a very limited number of genetic resources (Additional file 1: Table S1) in public databases. Newborn calves infected with BPV often develop digestive and respiratory issues, while pregnant cows often experience spontaneous abortions and stillbirths [5]. BPV infection has a prevalence of 83–100% in regions with cow herds around the world. The agent's prevalence across Africa is unknown, but it is likely widespread [36]. However, Egyptian cattle with respiratory distress were found to be infected with bovine parvovirus-3 (BPV-3) for the first time by Nagy et al., and the whole-genome sequence of BPV-3 exhibited 93.02% nucleotide identity with the reference virus (Additional file 1: Table S1) [25]. Calves from the United States (Maryland, South Dakota, Colorado and Oregon), Europe (England), Asia (Japan, China, and Korea), Oceania (Australia) and Africa (Algeria) all had bovine parvovirus (BPV) isolated from their feces, lymph nodes, tonsils, and conjunctival excretions at varying points in the infection course. According to historical records on BPV virus isolation from various nations [31], there is a wide range in the start of clinical signs such as diarrhea, conjunctivitis, and respiratory diseases, from as early as 1 week of age in calves to as late as 1 year of age [34]. Calves who were weaned at a younger age began excreting bovine parvovirus sooner, although this may be because they had a lower amount of maternally generated antibodies. An earlier-born calf without maternally acquired antibodies did not excrete parvovirus until four weeks after weaning. This demonstrates that most prevalence reports of bovine parvovirus are linked to calf diarrhea and that the virus causes economic losses in the cattle sector without causing any outward symptoms [29]. For instance, Lee et al. reported from Korean dairy farms that viruses (117/164, 71.3%) were the most common causative agent of calf diarrhea, and of these, 5.5% of the calves were infected with bovine parvovirus [21]. Concurrent infection with another enteric pathogen or other variables that stimulate intestinal epithelial proliferation may worsen the severity of the disease [24]. However, antibodies against nonbovine parvovirus antigens are unlikely to be detected in serological surveys in cattle as a result of cross-infection [6]. Cross-infection with nonbovine parvoviruses is therefore highly unlikely to be discovered in serological studies of cattle [6]. BPV strain VR-767 was also detected in clave fecal samples from the Heilongjiang, Jilin, Liaoning, and Inner Mongolia provinces using PCR [40]. An old serological study from North Queens Land, Australia, showed that calves could develop bovine parvovirus infection soon after weaning [10]. Despite the considerable evidence indicating the frequently endemic nature of bovine parvovirus in cattle, relatively little investigation has been made on the role of the virus in causing enteric disease [10]. There is a high prevalence of antibodies against bovine parvovirus 1 (BPoV-1) in naturally infected cattle across the globe [34], and all BPV-1 isolates have been found to be closely related to or identical to the prototype strain described by Abinanti and Warfield [1].

Physiochemically, parvoviruses are among the most stable viruses found in vertebrates [33]. Bovine parvoviruses, like most other parvoviruses, are extremely resistant to chemical and physical inactivating factors. The most reliable disinfection is achieved with 0.5% chlorox or ethylene oxide in the form of a nonexplosive mixture of 10% ethylene oxide and 90% carbon dioxide [25]. In addition, the virus can survive for up to six months when stored at − 20 °C [1]. Bovine parvovirus can maintain a pH ranging from 6.2 to 9. This virus can be isolated from liquid manure [26]. Hence, parvoviruses such as swine parvovirus [28, 30]), human parvovirus [2], canine parvovirus [11], and BPV [35] are currently playing a role in the nanoparticle vaccine sector, such as VLP chimeric vaccines against numerous infectious diseases. When used as a vaccination platform, virus-like particles (VLPs) can stimulate both humoral and cell-mediated immunity, leading to robust immune responses (Additional file 1: Fig. S1) [19].

According to the molecular architecture and genome organization, BPV is an autonomously replicating virus with a linear single-stranded DNA (ssDNA) genome of 5.5 kb flanked by nonidentical palindromic terminal hairpins, similar to other bocaparvoviruses [37]. Mature BPV virions are small, nonenveloped particles, 20–28 nm in diameter, with no known essential lipids, carbohydrates, accessory proteins or histones [17]. The absence of an envelope readily helps with in vitro assembly and the formation of soluble homogeneous noneVLPs that can be expressed in both eukaryotic and prokaryotic systems [37]. The whole genome encodes three ORFs (Additional file 1: Fig S2) [22].

To make an icosahedral viral capsid of 60 monomers, VP1 and VP2 capsid proteins can either co-assemble in a ratio of 5–95% or self-assemble alone [32]. The N-terminal region of VP1 is distinct for each species of parvovirus, with 411 and 227 residues for bovine and human parvovirus, respectively, in addition to VP2 amino acids. It is calculated that the mature virion has a buoyant density of 1.38 g/ml in a CsC density gradient [17]. Understanding how the capsid structure relates to the function of viruses provides a platform for recombinant engineering of viral gene delivery vectors for the treatment of clinical diseases [3]. Porcine parvovirus (PPV) VLPs produced by self-assembly through in vivo or in vitro methods could effectively display a foreign epitope [14, 39] and are good particles to design and develop chimeric epitope-based vaccines against PPV and FMDV [42] (Additional file 1: Table S2).

Despite the fact that parvovirals have good quality for such platforms, the potential of BPV VLP in displaying a foreign epitope for an epitope-based vaccination platform has barely been explored in comparison to that of other parvoviruses. Even less is known about the mechanisms by which BPV capsid proteins contribute to the successful production of VLPs in either prokaryotes or eukaryotes. No single experimental report on the role of VP1 of the BPV in the assembly and stability of VLPs is available. No previous study has evaluated the ability of recombinant BPV VP2 and VP1 VP2 Cap proteins to self-assemble into VLPs using an insect-baculovirus expression method. We believe that determining the stability and ease of generating BPV virus-like particles (VLPs) either from a single structural protein (VP2) or by combining both VP2 and VP1 proteins is critical to utilizing the VLPs of this virus as an anti-BPV vaccine and vaccine carrier.

## Materials and methods

**Fig. 1:**
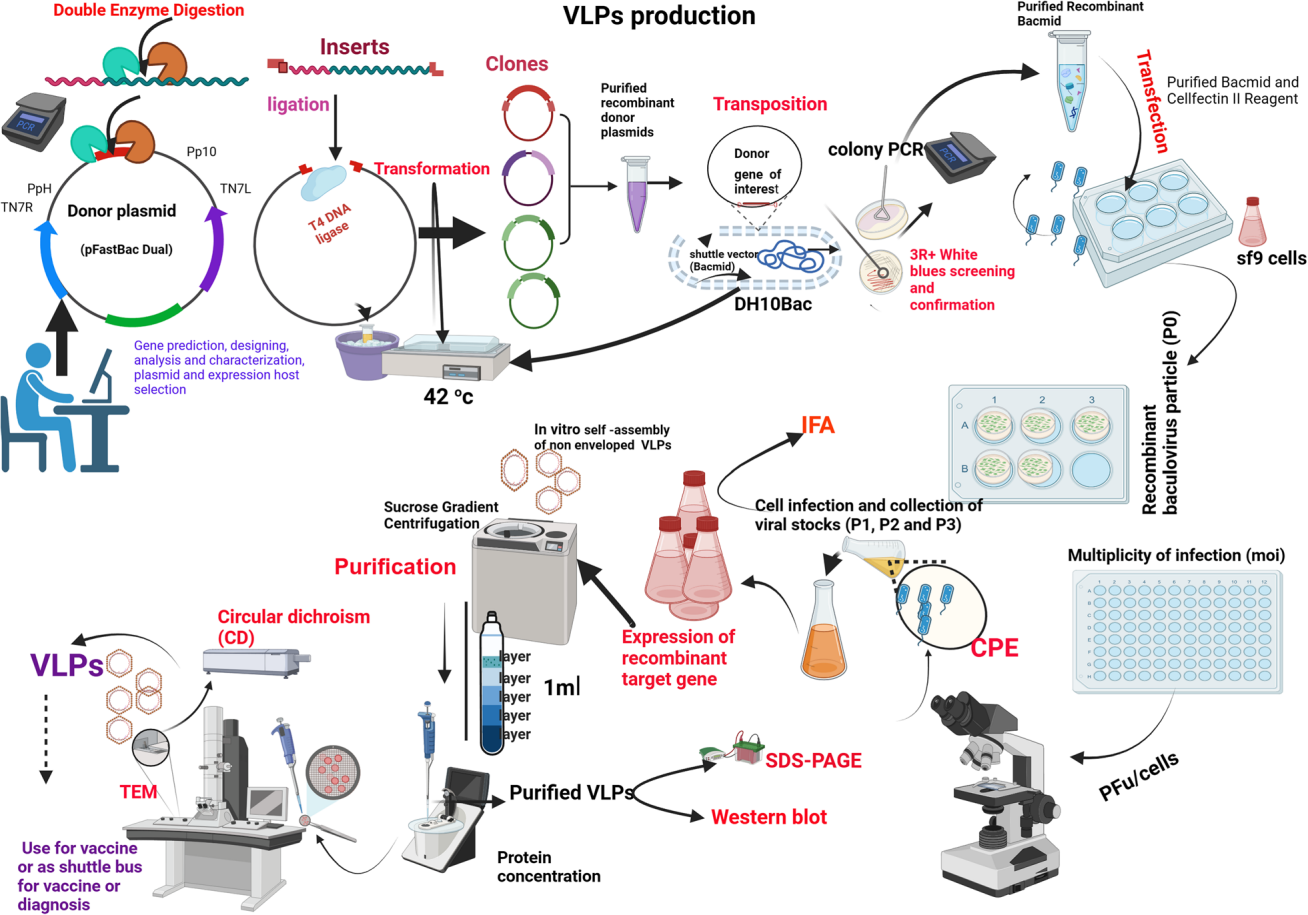
This diagram is a simplified representation of the research process as a whole

The aim of this study was to assess the role of VP1 in VLPs stability and assembly and to investigate whether a single structural protein (VP2) or a combination of VP2 and VP1 could be employed to successfully construct BPV VLPs. Furthermore, we experimentally verified the bioinformatically predicted physicochemical parameters (pH, ionic strength, and temperature) that most affect assembly yield and VLP stability. Details of the experiments are illustrated in the following architectural research process flow chart Fig. 1.

### Physico-chemical analysis of the capsid proteins of BPV (dry lab)

**Fig. 2:**
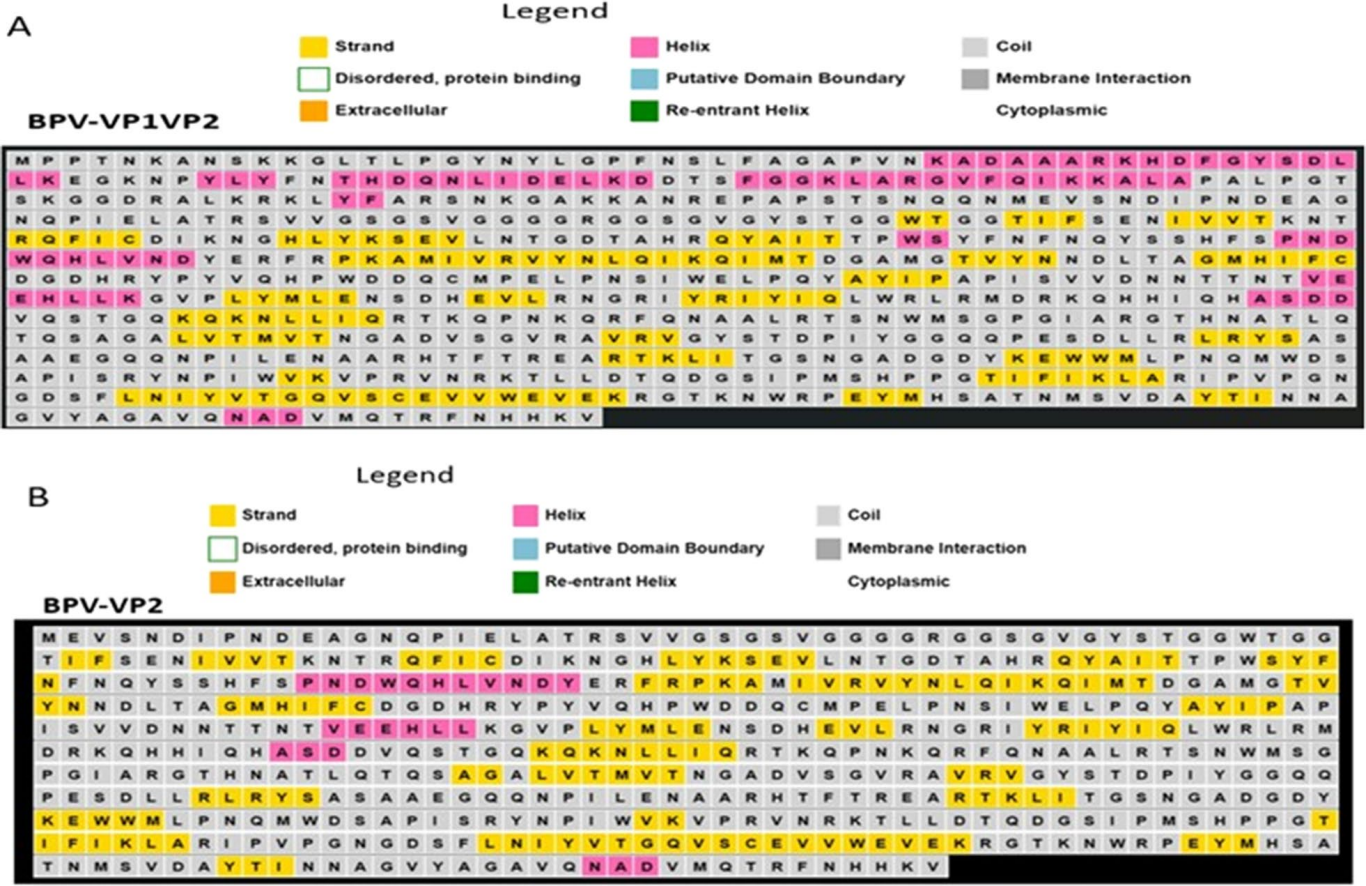
The graphic depicts PSIPRED 4.0's secondary structure prediction for the amino acid sequences VP1 and VP2 of BPV; **A** illustrates the various structural forms of the amino acid sequence of BPV-VP2, as indicated by colours, including helical, strand, random coil, and others. **B** represents the BPV-VP1VP2 amino acid sequence in various structural forms, as shown by different colours, including helices, strands, random coils, and others

Using the ProtParam website, we determined the expected physicochemical properties of the VLP protein sequences in Table 1. It was calculated that the resulting protein would have a molecular weight (MW) of 75.1 kDa for VP1 and 60.2 kDa for VP2 alone, with theoretical isoelectric point (pI) values of 9.27 and 8.18, respectively. Based on the pI, the protein is expected to have an alkaline pH. Both capsid proteins were estimated to have a half-life of 30 hours in human reticulocytes in vitro, 20 hours in yeast, and 10 hours in E. coli in vivo [16]. The anticipated instability index (II) for VP2 alone was 33.7, while for VP1VP2, it was 32.61, making the protein stable (II >40 implies instability). Expression of the protein was anticipated to result in an insoluble form (solubility score of 0.238) [38] and proteins with a higher scaled solubility value (value greater than 0.45) are predicted to be more soluble [20]. VP1VP2 and VP2 had aliphatic indices of 70.15 and 72.3%, respectively, indicating thermostability [20]. Aliphatic indices of 70.15 and 72.3% for VP1VP2 and VP2, respectively, were calculated, indicating that the capsid proteins are stable at high temperatures [20]. The predicted grand average of hydropathicity (GRAVY) was  − 0.407. A negative value indicates that the protein is hydrophilic in nature and can interact with water molecules [27]. A comparison of the predicted secondary structures of BPV VP1VP2 and BPV VP2 proteins (Fig. 2) reveals that they are very similar, with the exception of the number of alpha-helixes.

**Table 1:** Bioinformatic prediction of the physicochemical properties of the structural proteins of BPV that form VLPS

Capsid protein type	Nuc. Seq	aaseq	Instability index	Estimated half-life	Theoretical Pi	Hydropathicity	Total (− & +Vely) Residues, respectively	Aliphatic index
VP1VP2 (75.1KDa)	2022 bp	673aa	32.61 (stable)	30 h (mammalian reticulocytes, in vitro). > 20 h (yeast, in vivo). > 10 h (*Escherichia coli*, in vivo)	9.27	− 0.614	(Asp + Glu): 60 (Arg + Lys): 74	70.15
VP2 (60.2KDa)	1611 bp	536aa	33.73 (stable)	30 h (mammalian reticulocytes, in vitro). > 20 h (yeast, in vivo) > 10 h (*Escherichia coli*, in vivo)	8.18	− 0.560	(Asp + Glu): 49 (Arg + Lys): 50	72.3

### Chemicals, biological and synthetic biochemicals

Foreign and domestic commercial sources were used to acquire biological components and chemical reagents of analytical quality. DNA polymerase and T4 DNA ligase were acquired from TaKaRa Bio Inc. (Dalian, China), as well as the DNA marker, DNA restriction enzymes, E. coli DH10Bac cells, and Escherichia coli DH5 cells. Fetal bovine serum (FBS), GibcoTM Sf-900TM III SFM, and the pure LinkTM Quick Plasmid Miniprep Kit were all purchased from Thermo Fisher, USA). We used ExpiSf9TM cells that we originally purchased from Thermo Fisher, USA). Anti-His-Tag mouse monoclonal antibody and goat anti-mouse secondary antibody labeled with horseradish peroxidase (HRP) (Abcam, Cambridge, UK) Polyclonal Rabbit anti-DE-loop Antibody BPV-VP2 (SanQ Biotechnology Co.Ltd., Beijing) and HRP-labeled anti-rabbit IgG (from Sigma-Aldrich;St.Louis, Missouri, USA) were used.

### Polyclonal antibody

**Fig. 3:**
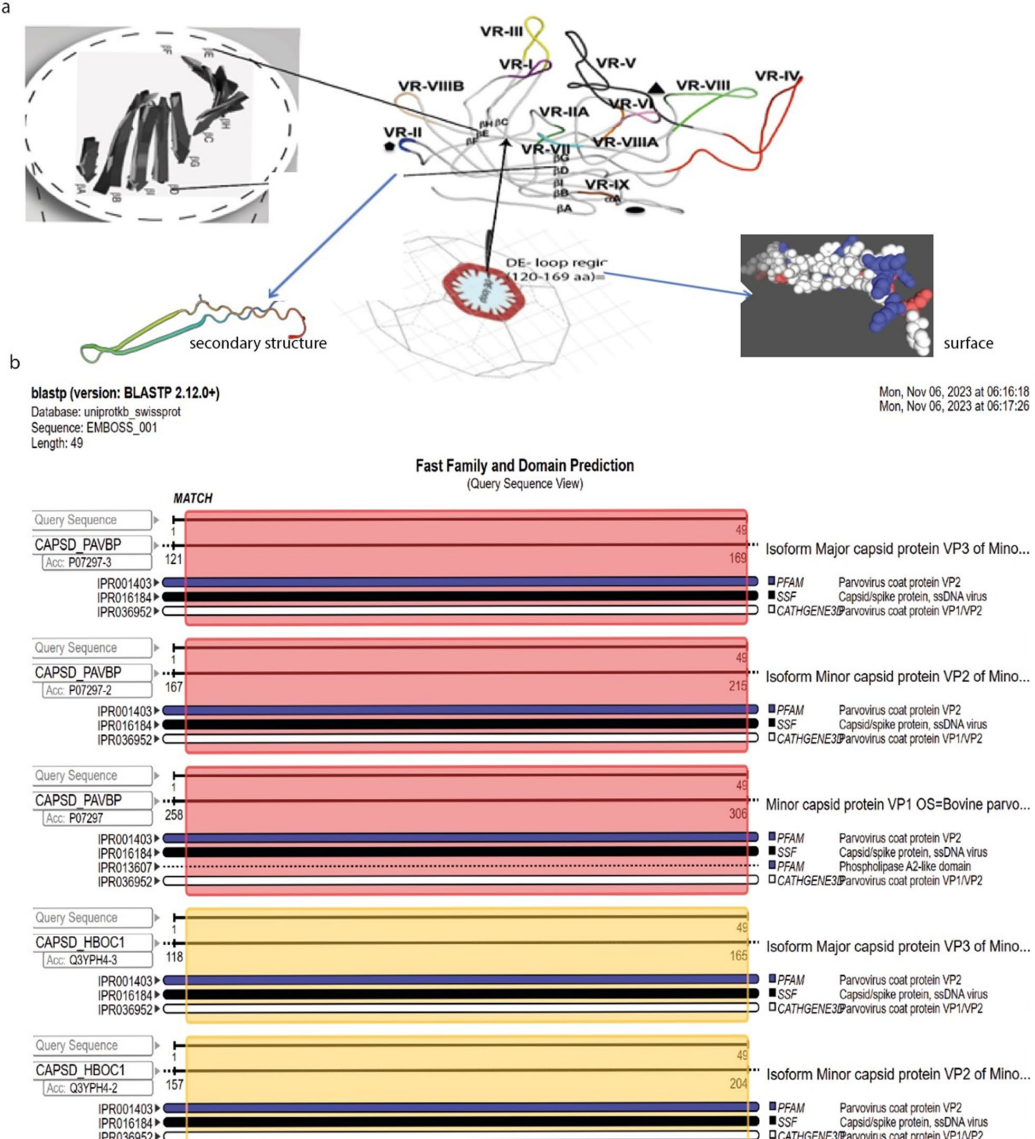
Tertiary structure and functional prediction of the DE-loop region of BPV-VP2; **a** schematic representation of the tertiary structure of BPV-VP2 is depicted above, specifically the location of the DE-loop between the D and E strands in the variable region (VRII) and surface topology. Blended and improved diagram adopted from [11, 32], **b** shows the result of the functional prediction of the 49 aa sequence of the bovine parvovirus type1

A DE loop joins two strands, βD and βE, and found on the surface capsid. Protein similarity analysis (job-ncbiblast-I20231106-061600-0533-17915353-p1m) shows that the DE-loop has 100% identity among all bovine parvovirus type strains but 73.5% sequence similarity with human bocavirus 1 type 1. The DE loop amino acids have been shown to be involved in VP1/VP2 externalization and antibody recognition [3]; Fig. 3). The IEDPA results showed that the antigenicity score calculated using the methods of Kolaskar and Tongaonkar was higher than the typical cutoff value of 1.013. Based on the results of the Bepipred Linear Epitope Prediction technique, the DE loop region is classified as a linear epitope with a prediction score greater than the cutoff value of 0.500. Therefore, the amino acid sequence corresponding to the DE-loop region was sent to SanQ Biotechnology Co. for peptide synthesis. The synthesized peptide was used to immunize rabbits, and the resulting serum was collected to obtain a polyclonal antibody (pAB) was produced.

### Construction of the recombinant pFastBac dual vector

**Fig. 4:**
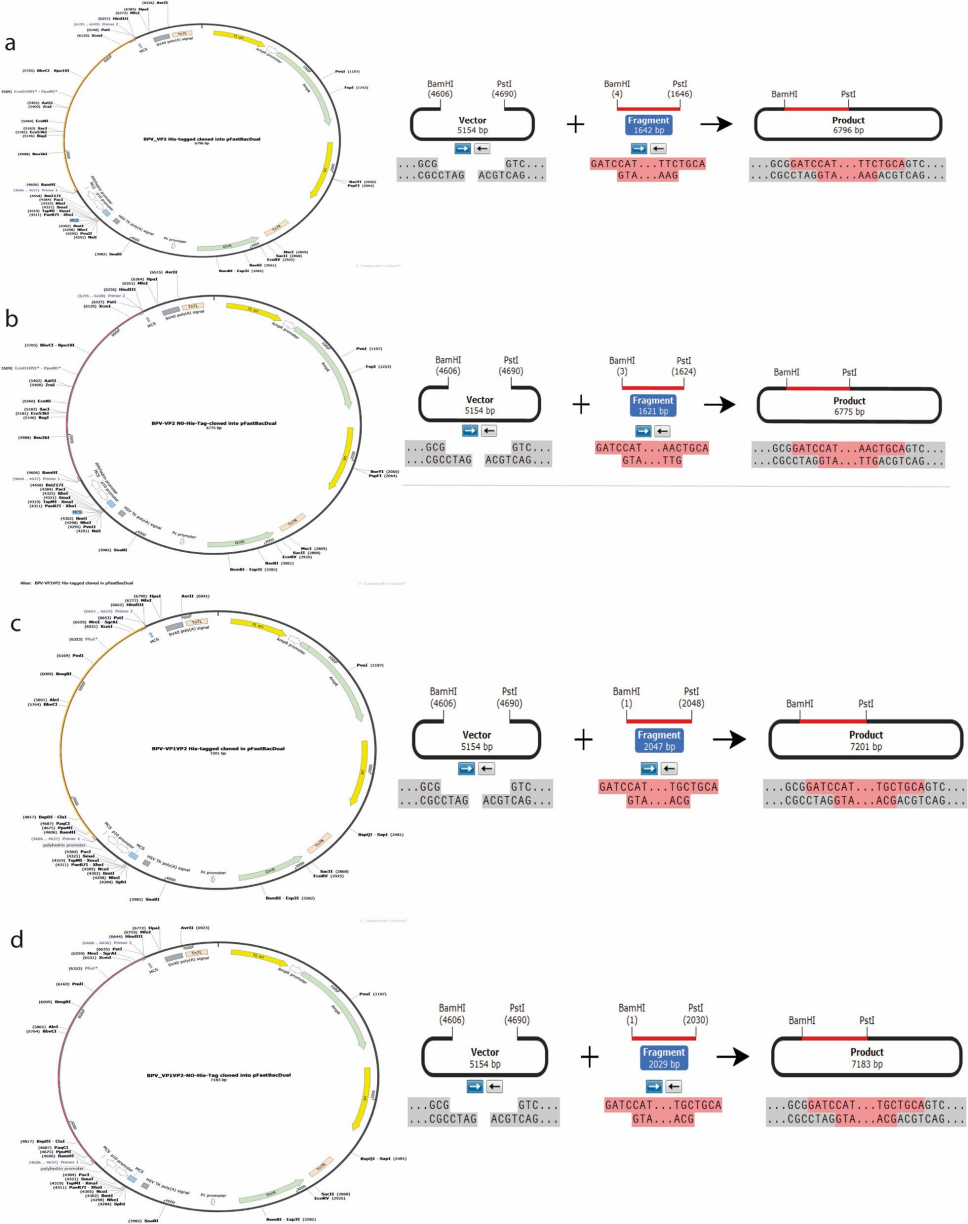
Insilco's building design was simulated using molecular architecture; **a** shows BPV-VP2 with His-tag **b** represents BPV-VP2 without His-tag **c** depicts BPV-VP1VP2 with His-tag **d** illustrates BPV-VP1VP2 without His-tag

In this study, we used recombinant bacmids BPV VP2 and VP1VP2 expressed in Sf9 cells to generate nonenveloped VLPs using the baculovirus vector expression system adhering to the protocols outlined in the Bac-to-Bac® system manual. Full-length capsid genes (BPV-VP1VP2 and VP2) were retrieved from GenBank under the accession number JN191349.1. In silico design by the Snap Gene program (https://www.snapgene.com/) was first applied to generate the recombinant BPV capsid proteins into vectors. The VP1VP2 (2022 bp) and VP2 (1611 bp) coding sequences were optimized in Vector Builder (https://en.vectorbuilder.com/tool/com) and amplified using primers with a His-tag and without a His-tag at the N-terminal fragment table. Four separate inserts were created using BamHI and PstI and cloned and inserted into pFastbacdual at matching restriction sites (Fig. 4). The anticipated completed architectural structures of the recombinant genes were used as a backdrop in the liquid experiment.

**Fig. 5:**
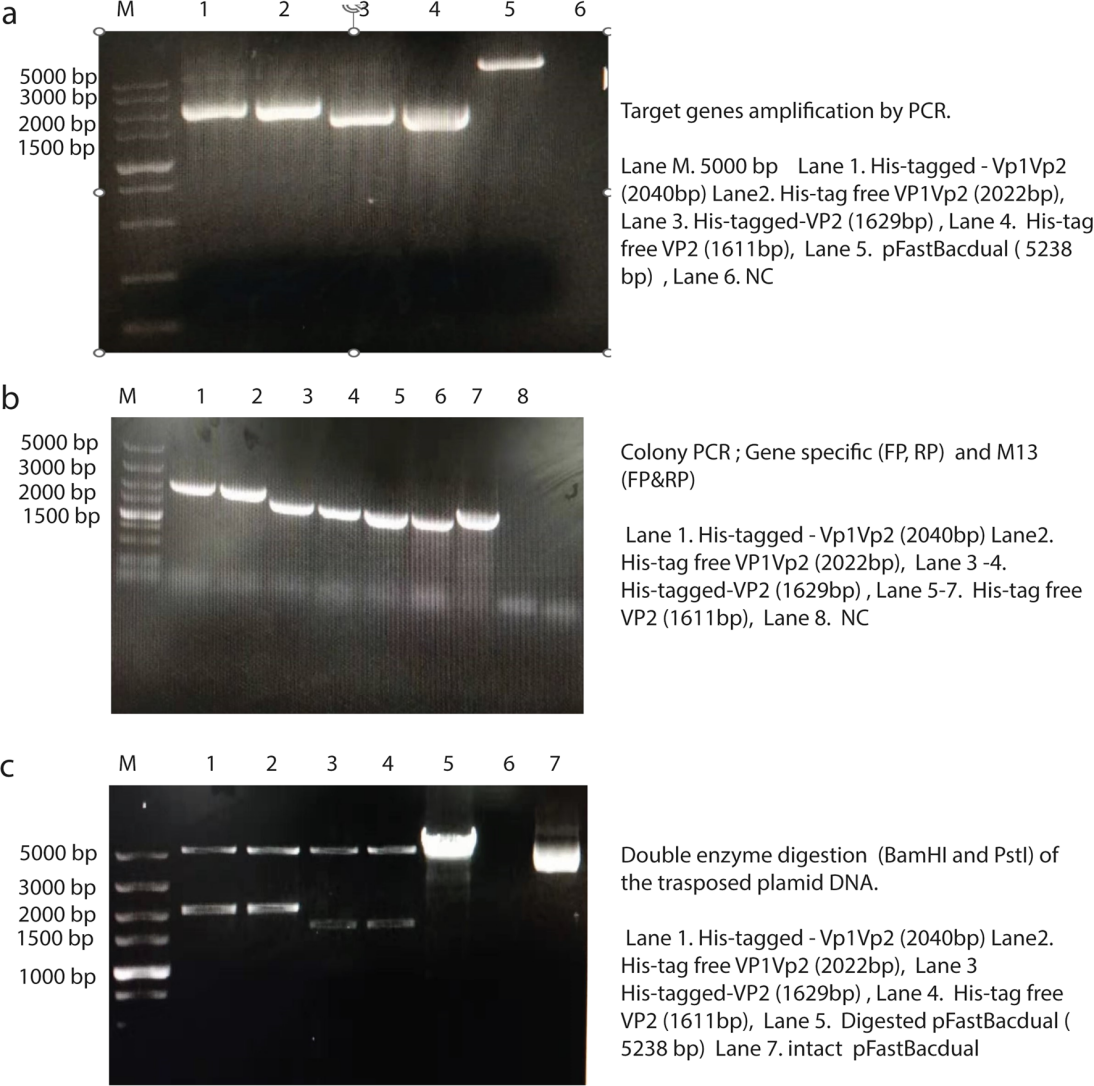
The results from PCR, colony PCR, and double enzyme digestion (**a**, **b** and **c**, respectively) were electrophoresed on agarose gels

Codon use optimization and gene synthesis of designed recombinants were performed at Gene Script (Piscataway, New Jersey, USA). Accordingly, the BPV VP2 and VP1VP2 segments were amplified (Fig. 5) using pairs of primers listed in Table 2 and structural genes built into the pUC vector as a template. The nucleotide primers were designed with restriction sites upstream (BamHI) and downstream (PstI). To generate the recombinant pFastBac BPV-VP2 and pFastBac BPV-VP1VP2 plasmids, the PCR products were digested with BamHI and PstI and then cloned and inserted into the appropriate restriction sites of the pFastBac dual vector. DNA sequencing and double enzyme digestion validated the insert of the recombinant plasmid. Transposition of the recombinant pFast donor plasmid into the bacmid needed the transformation of DH10BacTM. Bac-to-Bac® (Invitrogen Life Sciences) protocols were followed for both the transposition experiment and the subsequent transfection operations. To isolate recombinant bacmid DNA, only white colonies harboring the correct recombinant bacmid were chosen using the white‒blue screening method. To verify that the white colonies were indeed white, they were subcultured again on a new petri plate with the same antibiotics. A single pure white bacterial colony was inoculated into 2 mL of this LB medium, and the culture was left to grow overnight at 37 °C in a shaking water bath at 250 rpm. To purify and preserve (in 50% glycerol) the right recombinant DH10Bac for future use, a 2% inoculum (100 μL) was inoculated into 5 ml of LB medium containing 3R+ (penicillin, gentamicin and chloramphenicol) for purification purposes. The PureLinkTM HiPure Plasmid DNA Miniprep Kit (InvitrogenTM, Life Sciences, USA) was used to isolate high-quality Bacmid DNA (r-pFastBPV VP2 and VP1VP2) from the white colonies for transfection purposes. A polymerase chain reaction (PCR) employing M13 primers and gene-specific primers was subsequently performed on the recombinant Bacmid (pFastBPV VP2 and VP1VP2).

**Table 2:** The primers were designed using BamHI (upstream) and PstI (downstream) to amplify BPV-VP1VP2 and BPV-VP2 with and without His-tags

Primer type	Restriction Enzymes	Primer Sequences	Size of amplicon
FP- BPV- 1902	BamHI	5′CGGGATCCATGCCTCCCACCAACAAGGCTAACTC 3′	2400 bp & 1989 bp, respectively
RP-BPV-1902&1904 (His-minus)	PstI	5′ AACTGCAGTTACAGCACCTTGTGGTGGTTGAAACGG 3′	
FP-1904	BamHI	5′ CGGGATCC ATGGAGGTGTCTAACGACATCCC 3′	2022 bp
RP-1902&1904(His-plus)	PstI	5′ AACTGCAG TTAGTGATGATGATGATGATGCAGCACCTTGTGGTGGTTG 3′	1611 bp

### Transfection and expression of the recombinant protein

With the use of cellfectin® reagent, SF9 cells were transfected with purified and validated DNA inoculums of His-tagged r-BacVP2 and r-BacBPV1VP2 and His-tagged free r-BacVP2 and r-BacVP1VP2. Cells in a 6-well plate were monitored 24 hours after infection (pi) under an inverted microscope (EVOS) for any signs of CPE (Fig. 5). Hence, common symptoms of infection (CPEs, if any) were documented at 24, 48, 72, and 96 hours postinfection (pi). After confirming regular CPE and viability, we proceeded to harvest viral stocks. We optimized infection settings in Sf9 cells to obtain the highest possible production of VLPs. The MOI (5 pfu/cells) [7] of the recombinant baculovirus P2 stock of P1 and P3 stocks of P2 was found to be optimal for the infection of Sf9 insect cells and for the entire expression. For further expression, a pool of the viral stocks was clarified (at 500 rpm for 5 minutes), filtered (0.22 μm), and then stored at 4 degrees Celsius. Filtered P1 and P2 stocks were aliquoted and frozen at − 80 degrees Celsius in a black 1.5 milliliter centrifuge tube. After 4–6 days (cell viability <60–80% and cell size 18.5–21 µm), infected cells were frozen and thawed three times at intervals of 3–4 hours and centrifuged at low speed (10,000 r/min) for 30 minutes at 4 °C to remove the cell debris. The recombinant proteins that had been expressed were then harvested from the supernatant.

### VLPs purification methods

Overnight, the supernatant was stirred on multipoint magnetic stirrers (Thermo Fisher, USA) with 8% (8 g/1 L) polyethylene glycol 8000 and 0.5 M NaCl (0.5x 1Lx58.44 = 29.22 g) in a 4 °C cold environment. After 45 minutes of centrifugation at 12,000 rpm and 4 degrees Celsius, the pellet (precipitate) was collected and then resuspended in cold, sterile 1xPBS. Debris and insoluble proteins were then removed by centrifugation at a low speed. Ultracentrifugation with a 10–40% sucrose gradient at 36,000 rpm for 3 hours at 4 °C in a Beckman Optima L100XP centrifuge using a SW32 Ti rotor (Beckman Coulter, Thermo Fisher Science, USA) was used to further purify the clear supernatant, as described by two other authors [7, 18]. Ultracentrifuged VLP protein content (mg/ml) and purity (260/280 wavelength) were determined using a UV‒Vis spectrophotometer (Thermo Scientific NanoDropTM One, Rockford, USA) throughout 11 ml, with 1 ml in each 1.5 ml centrifuge tube. IFAT, SDS‒PAGE, Western blotting, and TEM negative staining were used to determine the protein's intracellular location, expression level, purity, immunogenicity, and VLP (rec-VP2 and rec-VP1VP2) integrity.

### SDS‒PAGE and western blotting

Undiluted pure recombinant proteins (0.35 mg–5.57 mg/ml) were separated by 12% SDS‒PAGE, and their protein bands were visualized using Coomassie Brilliant Blue R250 (Sigma‒Aldrich, St. Louis, USA). For western blotting, purified proteins were diluted to 20–30 µg/ml, separated by 12% SDS‒PAGE, transferred to PVDF membranes, and blocked with 5% (w/v) nonfat milk for 1 hour at room temperature (RT). The immunodominant conserved DE-loop at the N-terminus of the VP2 BPV protein bioinformatically predicts immunogenic function [18]. To our understanding, we synthesized this region for the first time as a polyclonal antibody for target protein detection. To detect the four recombinants, we used a primary antibody of rabbit anti-DE-loop Polyclonal IgG (1:5000) (SanQ Biotechnology Co.Ltd., Beijing) and a secondary antibody of mouse anti-rabbit IgG (1:5000) (SIGMA), with three washes in phosphate-buffered saline-Tween solution (PBST) at five-minute intervals between each step. In addition, mouse anti-His-tag monoclonal antibody (diluted at 1:2000; Abcam, Cambridge, UK) and rabbit anti-mouse IgG antibody (diluted at 1:5000) were employed to detect His-tagged recombinants using the same methods described above. Recombinant protein bands were characterized using an ECL machine (GE IA600, AmershamTM, He Nan De Quan Xingye, Co. Ltd., China) and a chemiluminescence kit (Yeasen Biotechnology Co. Ltd, Shanghai).

### IFAT (indirect immunofluorescence assay)

Sf9 cells (2x10^6^) were transfected with four BPV-VP2 recombinants at m.o.i of 5, bacmid alone as a positive control, and mock-infected cells as a negative control in a 6-well plate with a cell density of 1x10^6^. At 24, 48, 72, and 96 hours postinfection, cells were fixed with 4% paraformaldehyde in PBS pH 7.4 for 10 minutes at room temperature to assess the level of expression of all recombinants. After blocking for an hour with 1% BSA/milk at 37 °C, all wells were rinsed three times for five minutes in PBS. After incubation with DE-loop pAb (1:5000) specific for BPV-VP2 protein, the cells were washed. Red fluorescent protein (RFP) Alexa Fluor® 594-conjugated anti-rabbit IgG antibodies were diluted to 1:800 and incubated in the dark at room temperature for 60 minutes. Fluorescence microscopy (EVOS FL Cell Imaging System, Thermo Scientific, USA) was used to observe the reaction.

### Transmission electron microscopy (TEM)

Lysates from rBac-VP1VP2- and VP2-infected Sf9 cells were separated by sucrose density gradient centrifugation, as described previously [23]. The samples were diluted to a concentration of 0.2–0.3 mg/L (100 ng). Approximately 10 µL of the purified fractions were incubated for 5 minutes at room temperature on a formvar-carbon film-coated copper grid (400 mesh, Pelco, CA, USA). The grid was dyed with 10 µL of 3% ammonium phosphotungstic acid (Ph = 6.5) after being rinsed three times in PBS. The grids were prepared for analysis by carefully removing any excess sample, PBS, and dye with filter paper or tissue paper. A Hitachi Limited (Japan) HT-7700 120 kV TEM was used to observe the assembly of recombinant BPV virus-like particles (VLPS).

### Stability and secondary structure analysis of VLPs of BPV (VP1+VP2) and (VP2)

VP1 is a small, structural, and nonessential protein of BPV. This may help keep the BPV particles stable. To determine what role VP1 plays in keeping BPV particles stable, two types of VLPs were studied at various temperatures and pH levels. These were the VP1 and VP1+VP2 VLPs Two kinds of VLPs were treated with different phosphate buffers (pH 4.5 to pH 8); the secondary structure was then analyzed by far UV circular dichroism (CD) spectroscopy. The percentages of the secondary structures α-helix, β-sheet, and β-turn, as well as the percentage of coil in VLPs (VP1+VP2) and (VP2) after being treated with different PBS (phosphate buffers, pH 4 to pH 8), were analyzed using USB circular dichroism (CD) spectroscopy. The thermal stability of VLPs produced in PBS at 4 °C was assessed to determine the integrity of the particle or if morphological disruption or changes existed while exposed to different temperatures (20 °C, 40 °C, 60 °C, and 80 °C) for 30 minutes. Changes in the morphology or integrity of the VLPS while incubating VLPs at different temperatures were also analyzed by TEM.

## Results

### In silico construction of the bovine parvovirus recombinant bacmid

Here, a total of four candidate clones of BPV-VP2 and VP1VP2 proteins were generated using an in-silico technique (Fig. 4). For cloning, we recommended a codon-optimized sequence and modeled primers for PCR amplification of the inserts, cloning into the vector, and expression in insect hosts.

### Generating the recombinant bacmid and the production of recombinant baculovirus

**Fig. 6:**
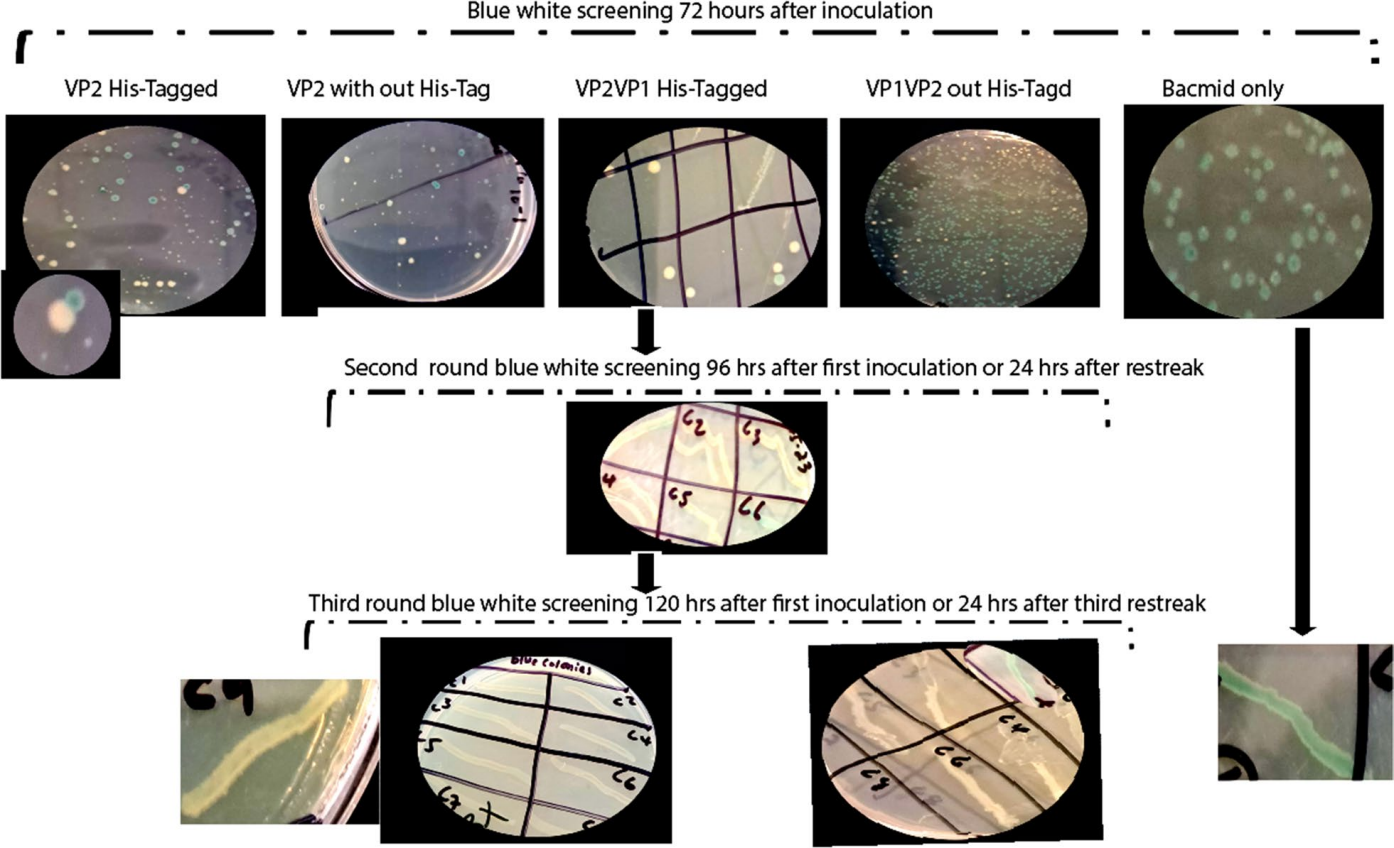
Displays the blue and white screening processes that were used in our investigation. The details of the experiment are labelled on the picture. The Blue‒White Screening of Transposed DH10BacTM competent cells after three days of growth in a 37 °C incubator is shown in these pictures. The first upper panel; on the third day, only white colonies were restreakd to new petri plates to prove that the transformed competent cells with the desired inserts were pure. Small colonies of both blue and white started growing after 48 hours, and by 72 hours, they were large enough to be differentiated. In the second panel; Individual white colonies that were originally white were still white after being restreaked for in the second round of screening after 24 hours. In the third panel; the white colonies that were chosen in the second round of screening remained white after restreaking and incubation for 24 hours in the third and final rounds. In the Left edge of the upper and third panel; shows colonies containing only bacmid; after three rounds of screening, the Bacmid alone (negative control) still showed a blue color

Sequencing and double enzyme digestion validated the cloning of the recombinant bacmid DNA into the pFastdual donor vector (labeled rec-BPV-VP1VP2 and rec-VP2 with/without His-tag; Fig. 5c). As shown in Fig. 6, the DH10BacTM transposed bacmid was successfully isolated in white colonies following successive subculturing. The recombinant DNA that was amplified by PCR using M13 primers and gene-specific primers was determined to be in the correct orientation (Fig. 5).

### Transfection and expression of the recombinant protein

**Fig. 7:**
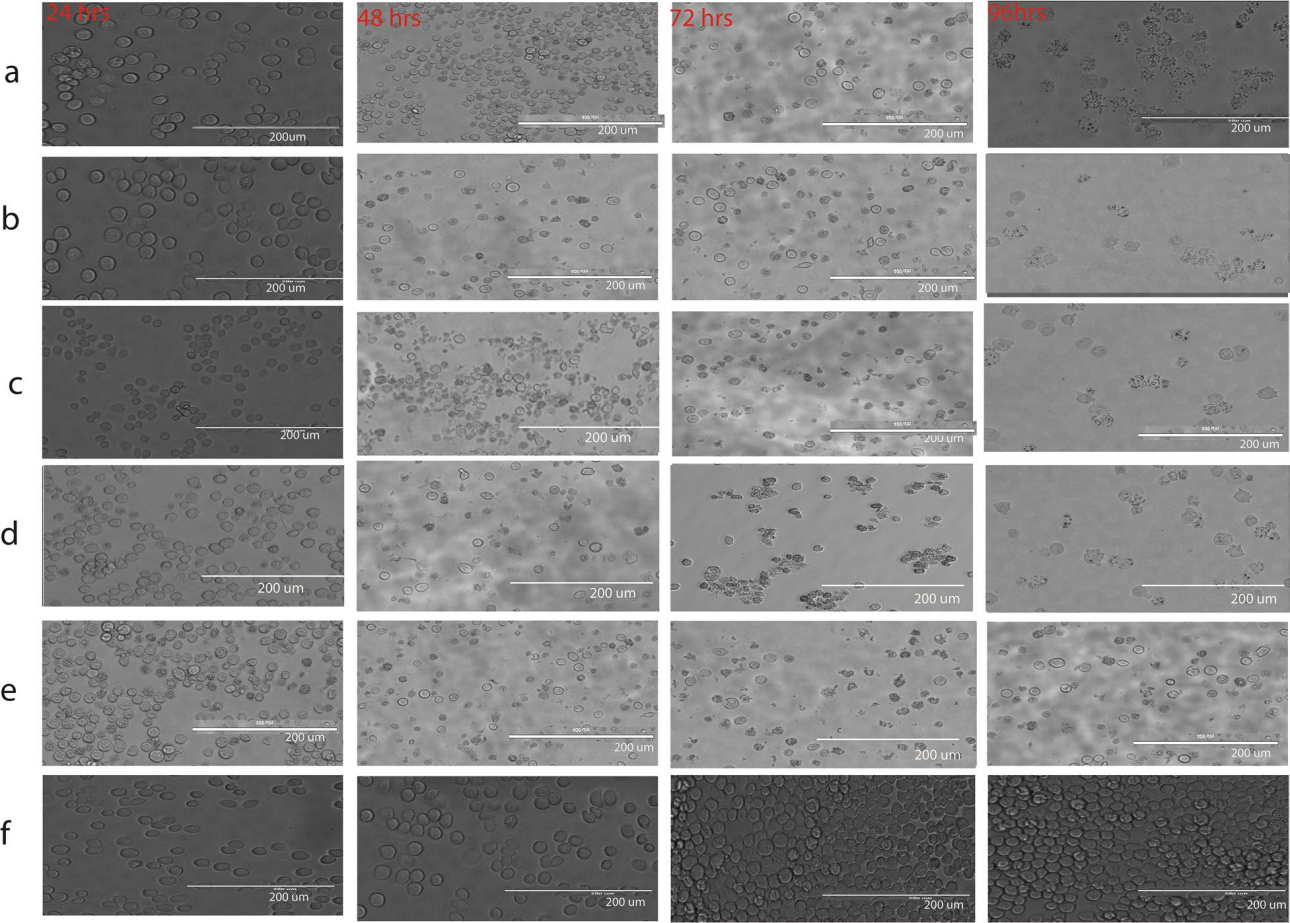
Four recombinant BPV-viral clones (VP1VP2 His-tagged, VP1VP2 without His-tag, VP2 His-tagged and VP2 without His-tag) were transfected into Sf9 cells using freshly recovered bacmids. For transfection, we employed cellfectin (2 ng Bacmid/8 ng cellfectin) to create recombinant baculovirus stocks (**a**–**d**). **a** VP2 His-tagged (BPV-VP2 with 6xhistag), **b** VP2 without His-tag (BPV-VP2 without 6xhistag), **c** VP1VP2 His-tagged (BPV-VP2VP1 with 6xhistag), **d** VP1VP2 without His-tag (BPV-VP2VP1 without 6xhistag), **e** PfastBacdual (positive control) **f** Mock (cell only). All recombinants showed an increase in cell width by 16.79 to 18. At a thickness of 79 µm 24 h after infection, 48 h pi cells were full of nuclei and no longer dividing, and 72- and 96-h pi cells showed a granular appearance (20.23 µm), detachment, and lysis. However, negative controls (cells only) appear spherical and somewhat granular (of normal size) during all phases of infection. 96 h in, it had a firm attachment on the surface, was vigorously dividing, and had become overly confluent

Following the transposition reaction, all recombinant bacmid DNA with a high molecular weight was isolated. Successful transfection of five recBPV-isolated bacmid DNA clones (VP1VP2 with His-Tag, VP1VP2 without His-Tag, and VP2 with His-Tag, VP2 without His-Tag and pFastBac Dual) into Sf9 cells using cellfectin® Reagent according to the Bac-to-Bac® technique was achieved. Sf9 cells were visually evaluated daily for evidence of infection, including enlarged cell width, larger than normal nuclei, a halt in cell development, a granular appearance, detachment, and cell lysis during early, late, and very late infection stages (Fig. 7). After infection, cells were analyzed for cytopathic effects (CPE) using a cell imaging system (EVOS Thermo Fisher, USA) at 24, 48, 72, and 96 hours post infection (Fig. 7). Approximately 72-96 hours after transfection, when the cells begin to exhibit indications of lysis (viability 62–73%), approximately 2 mL media containing the recombinant virus (p1 virus stock) is removed from each well and placed into sterile 15 mL snap-cap tubes. The viral stocks were purified to a clear supernatant by centrifugation at 500 Xg (≈ 2100–2300 rpm) for 5 minutes. The virus was purified by passing the supernatant through a (0.2 μm) membrane filter before subsequent infections. High-titer p1, p2, and p3 viral stocks of 1904H+, 1904H-, 1902H+ and 1902H- and pFastBacdual were generated by infecting cells with p0 at the MOI 0.05 pfu/cell recommended by the Bac-to-BacTM baculovirus expression system user handbook. In this study, expression of the target protein was achieved using P2 and P3.

### Purification, Western blotting and transmission electron microscopy

**Fig. 8:**
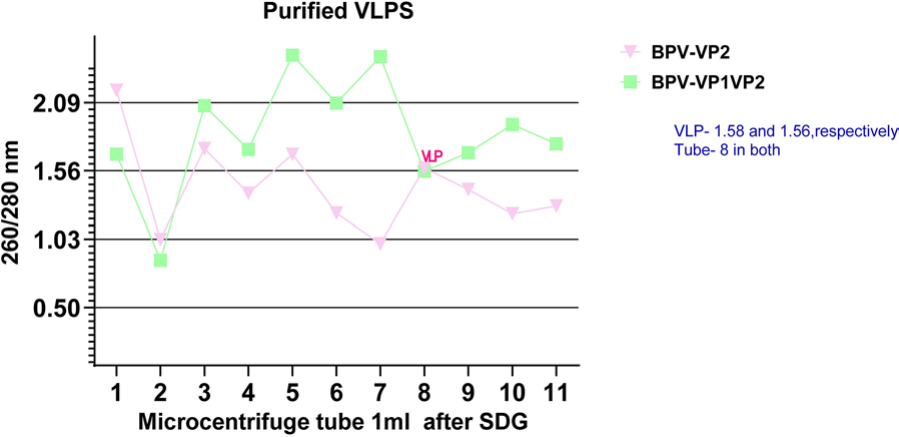
This graph shows the absorbance value at 260/280 of the purified protein where our BPVVP2 and BPV-VP1VP2 VLPs after concentration by PEG and Sucrose density Ultracentrifugation

**Fig. 9:**
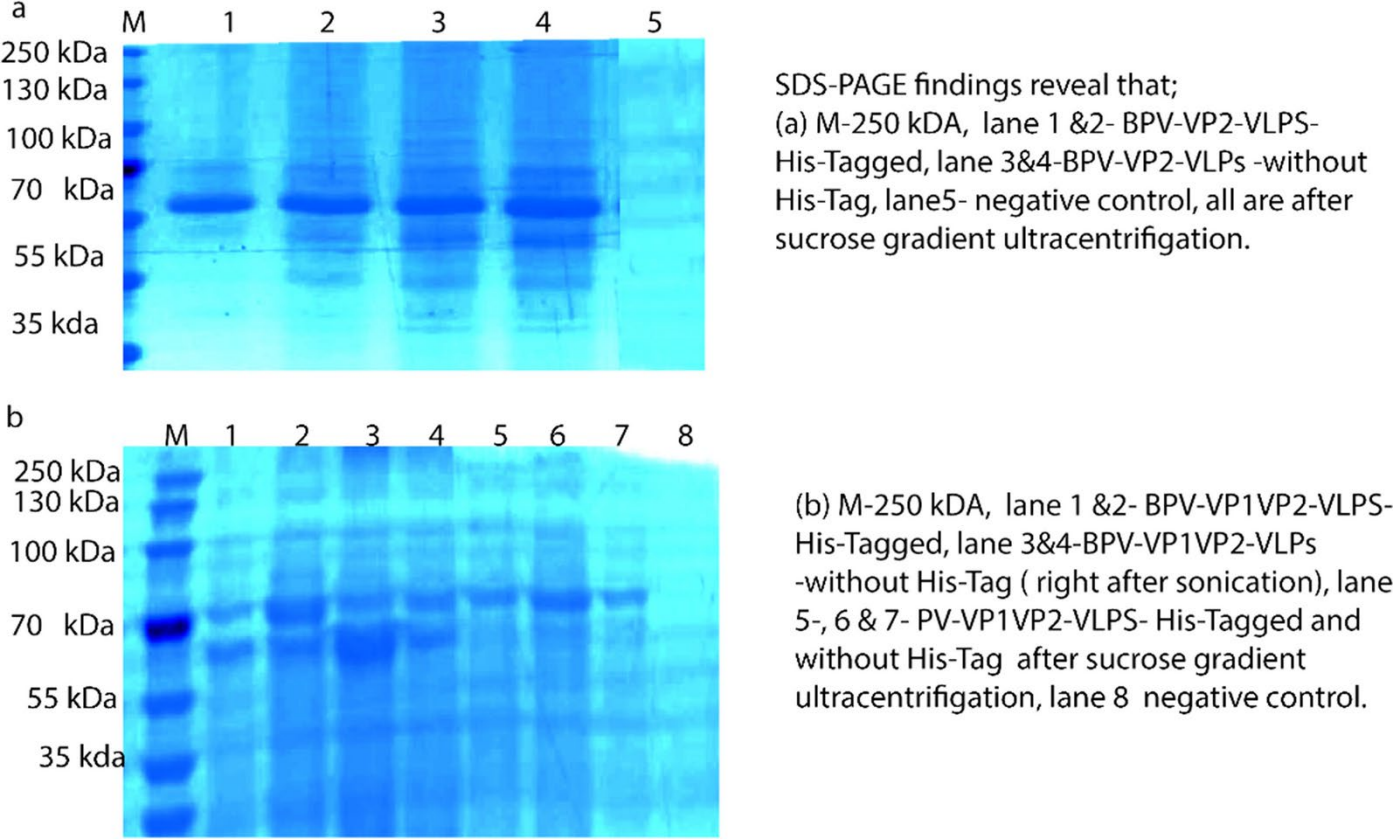
SDS‒PAGE analysis of purified and unpurified recombinant BPV-VLPs. The left panel of each figure explains the results in details; **a** shows the SDS-Page results of BPV-VP2 purified protein with and without His-tag; **b** demonstrates BPV-VP1VP2 of BPV-VP2 purified protein with and without His-tag

**Fig. 10:**
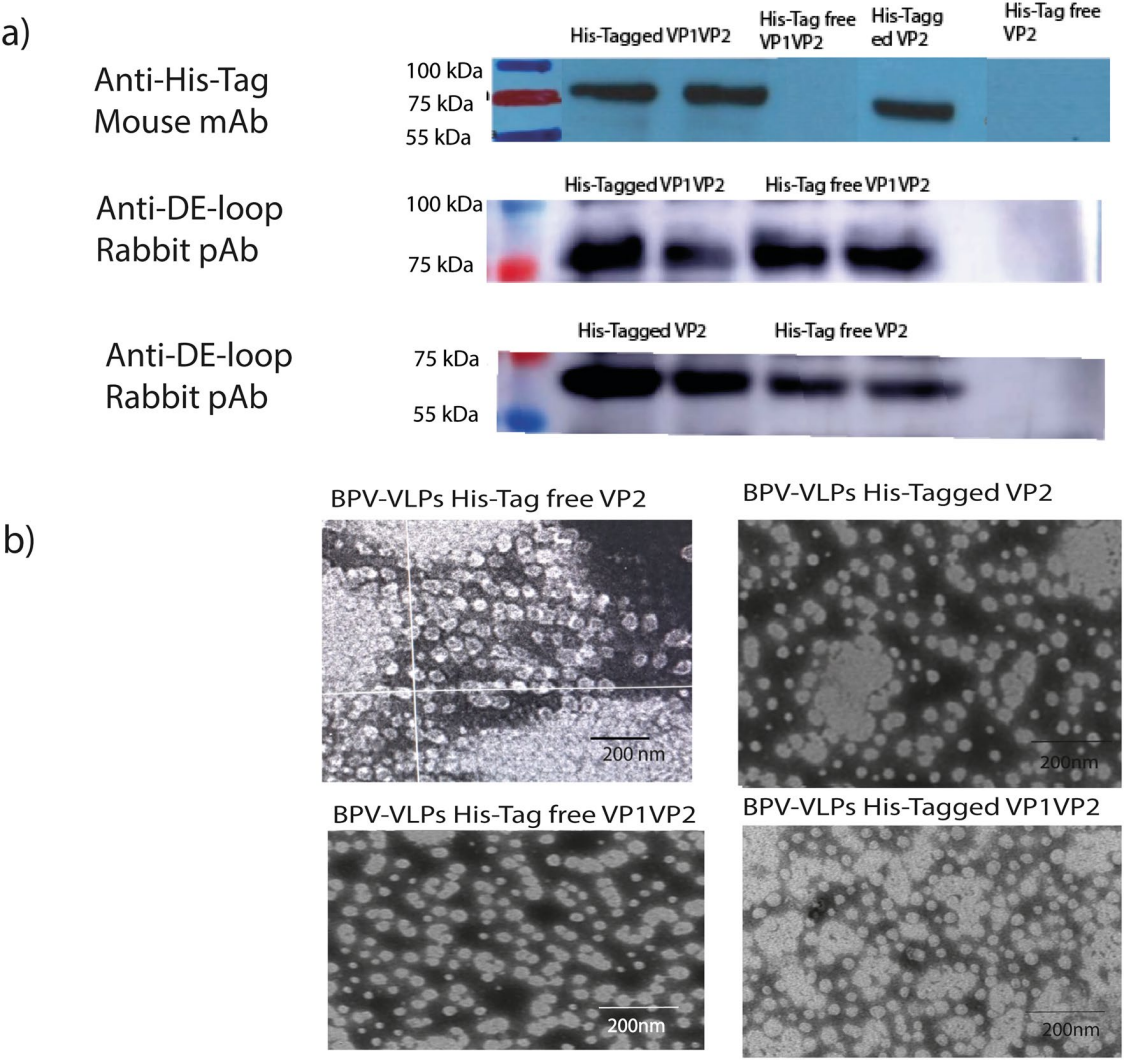
Western blot analysis of purified recombinant protein and negatively stained VLPs under a transmission electron microscope, **a** Purified VP1VP2 and VP2 recombinant proteins, with molecular weights of 75 kDa and 60 kDa, were recognized by an anti-His-tag monoclonal antibody and anti-DE-loop polyclonal antibody derived from BPV-VP2, **b** This TEM image demonstrates that BPV capsid proteins, both VP2 alone and VP1VP2, self-assembled to form ≈25 nm VLPs with uniform morphology

The sucrose cushion and sucrose density gradient procedures yielded very pure and uniform VLPs. High concentration of VLPs were found at the 8th tube with absorbance at 260/280 of 1.54–1.58 (Fig. 8) in the nanodrop one (Thermo Scientific, Rockford, USA). Detectable levels of VP1VP2 and VP2 proteins were expressed in Sf9 cells as determined by SDS‒PAGE and western blot examination of baculovirus-infected cell lysates (Figs. 9 and 10). Electron microscopy analysis of the purified products showed that the particle structure of all BPV VLPs, at approximately 25 nm (Fig. 10), was consistent with that of the natural BPV particles.

### Indirect immunofluorescence assay (IFA)

**Fig. 11:**
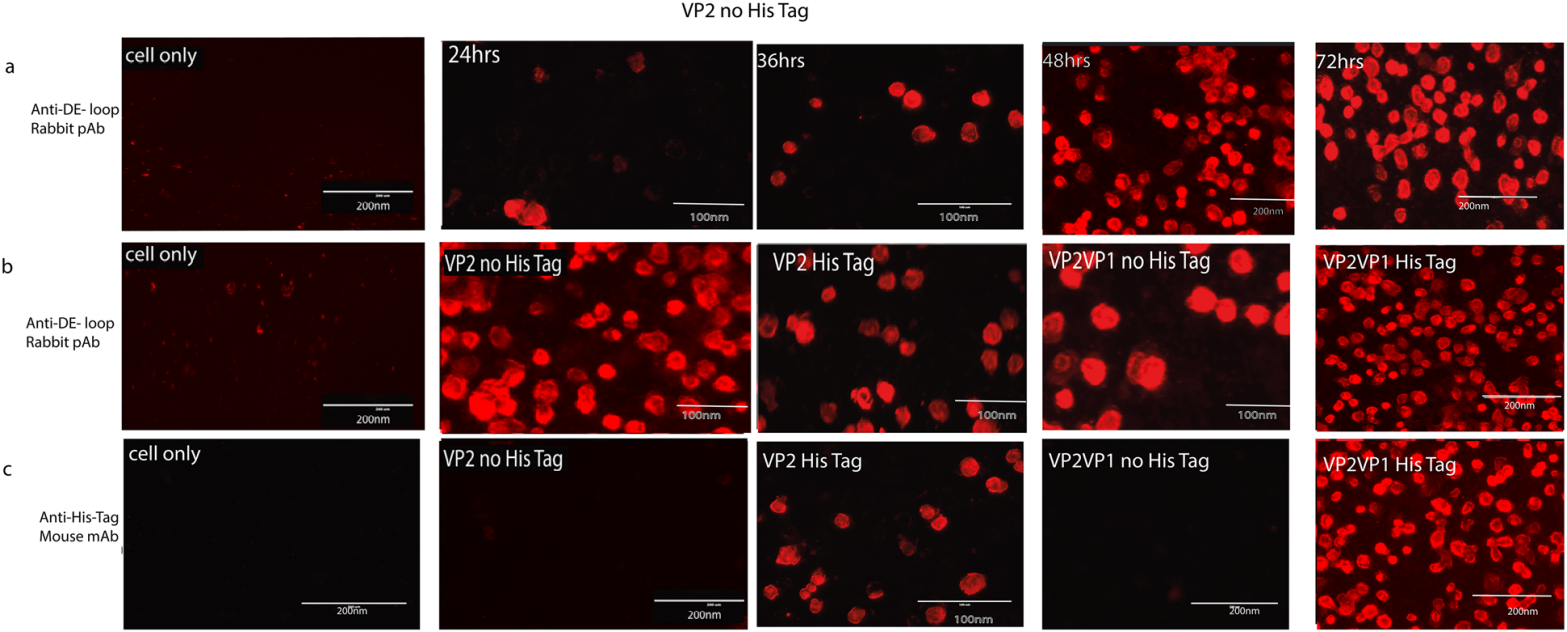
Illustrates the expression levels of recombinant proteins and their specific reactions with different antibodies, specifically VP1VP2 and VP2 alone, in Sf9 cells that were infected with recBacmid BPV: VP2 and recBacmid BPV: VP1VP2 at different time points. In all the panels the first in the left column represents the mock (cell only) as negative control, **a** At 24-, 48-, and 72-h post-transfection, the first panel displays the cells infected only with recBacmid BPVVP2 without His-tag, together with the level of expressed VP2 recombinant protein identified with pAb; **b** The second panel used anti-DE-loop rabbit polyclonal antibodies to identify His-tagged and His-Tag free VP1VP2 and VP2 protein expression levels; **C** The third panel displayed the expression levels of proteins from His-tagged and His-Tag free VP1VP2 and VP2 that were detected with anti-His-Tag mouse monoclonal antibodies. These proteins were His-tagged and His-Tag free. In this experiment, RFP (red fluorescence antibodies) Alexa-Fluor®-594-conjugated anti-rabbit IgG and antimouse secondary antibodies were used. Generally, the reaction in the fluorescence microscope clearly shows that the rec-proteins from different designs vary in expression at different time points, and the mAb and pAb are highly specific in the detection of the target proteins

The objective of this experiment was to assess the expression level of the VP2 recombinant protein in infected cells. The cells were infected with a multiplicity of infection (m.o.i.) of 5, and the expression levels were measured at four time points: 24, 36, 48 and 72 hours postinfection. The expression levels of recombinant VP1VP2 and VP2 alone proteins in Sf9 cells infected with recBacmid BPV: VP2 and recBacmid BPV: VP1VP2 are shown in (Fig. 11). The protein expression of VP1VP2 and VP2, both with His-tags and without His-tags, was assessed.

### Stability of VLPs of BPV (VP1+VP2) and (VP2)

**Fig. 12:**
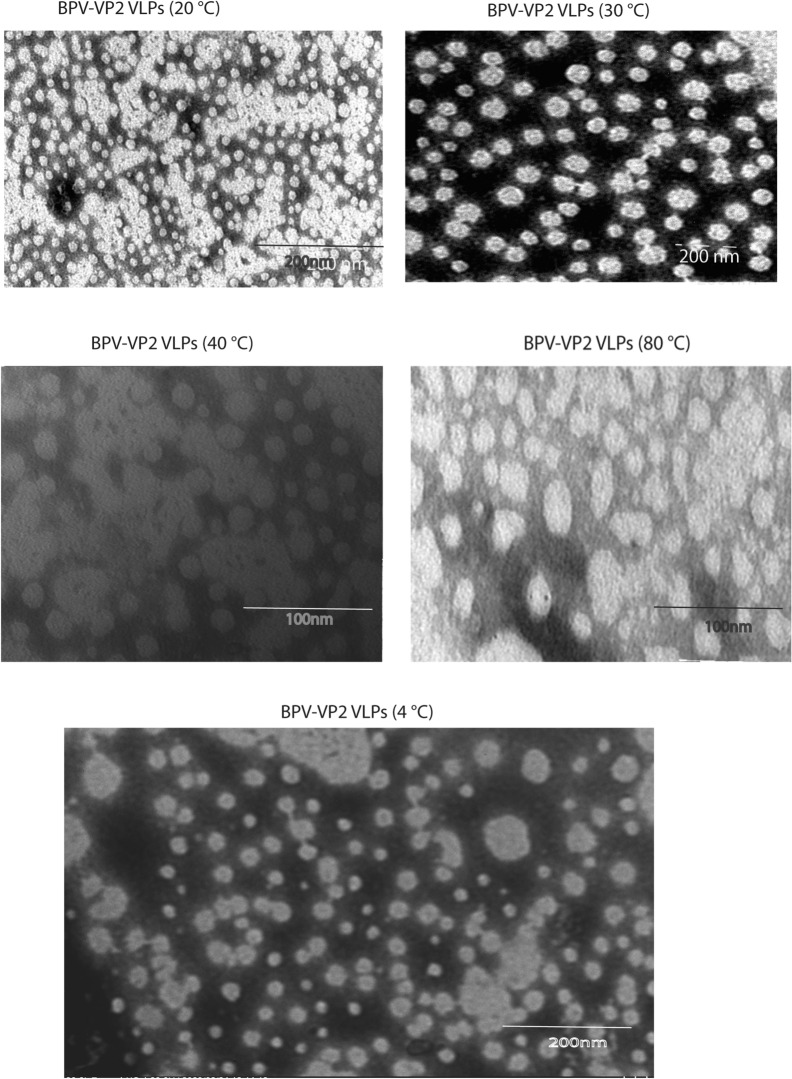
Transmission electron micrograph analyzing the morphological alterations and structural integrity of two types of VLPs following incubation at 20 °C, 30 °C, 40 °C, and 80 °C for 30 min to assess the thermal stability of BPV-VP2 VLPs in PBS

Based on the (circular dichroism) CD analysis findings of secondary structure percentages (Table 1), neither the BPVVP1VP2 nor the BPV VP2 VLP proteins exhibited appreciable changes in their secondary structure between pH 5 and pH 8. Hence, the secondary structure stability of the two kinds of VLPs was comparatively similar in buffers at pH 5 to pH 8 and corresponds to the results of bioinformatically predicted stability of VLPs (BPV-VP2 and BPV-VP1VP2) (Fig. 12). From pH 5 to pH 8, the percentages of β-sheets and β-turns are identical for VLPs (VP1VP2) and VLPs (VP2); however, the percentages of α-helices and random coils are slightly different (Table 3). The thermal stability of VLPs showed that the integrity of the particle remains intact at temperatures up to 80 °C. Slight morphological disruption or changes existed while exposed to the higher temperatures as shown in (Fig 12.).

**Table 3:** Experimental comparison of secondary structural proteins of the BPV VLPs

		BPV-VP2 VLPS			BPV-VP1VP2 VLPS		
		PH 5	PH7	PH8	PH5	PH7	PH8
αHelix	At 195–260 nm	11.40%	16.20%	14.50%	11.60%	16.9%	16.08%
βsheet		43.4%	38.2%	38.5%	43.4%	38.20%	38.50%
βturn		17.6%	17.30	17.20%	17.6%	17.32%	17.50%
Rand coil		31.2%	29.80	29.20%	31.30%	30.10%	29.40%
Total		103.6%	101.6%	99.30%	103.8	102.5	101.4

## Discussion

BPV infection has a prevalence of 83%-100% in regions with cow herds around the world. This demonstrates that most prevalence reports of bovine parvovirus are linked to calf diarrhea and that the virus causes economic losses in the cattle sector without causing any outward symptoms [29]. More importantly, they have the potential to produce visible to silent economic catastrophes in the livestock business. However, there has been no description of cattle vaccination attempts as preventive measures and treatments available for this virus and bocaparvoviruses [11]. More research into developing vaccines against BPV and other enteric viruses is likely to be necessary as we gain a better understanding of the infections and their potential pathogenicity, particularly as they pertain to the prevention of reproductive problems [25]. If implemented, this strategy might help reduce parvovirus and other enteric virus-induced infections in calves throughout their newborn period.

The BPV molecular architecture contains ORF3, which encodes 60 copies of VP1 and VP2 and plays a crucial role in the assembly of VLPs. Human baculovirus type 1 (HBoV1), on the other hand, is thought to produce 95% of its VLPs exclusively from VP2 [32], which resembles the structural proteins of BPV. In a similar manner to BPV, at least 60 copies of Bocaparvovirus VP2 organized into VLPs, as demonstrated by Guard et al. [14]. However, the effectiveness of generating BPV virus-like particles (VLPs) from a single structural protein (VP2) and by combining both VP2 and VP1 amino acid sequences and the function of VP1 in the assembly and stability of VLPs has not yet been studied. As a result, the future potential of BPV VLPs in displaying foreign epitopes in epitope-based vaccine platforms has barely been explored compared to that of other parvoviruses. Therefore, understanding how the capsid structure relates to the function of parvoviruses provides a platform for recombinant engineering of viral gene delivery vectors for the treatment of clinical diseases [3].

In the fight against infectious diseases in both animals and humans, VLPs have recently come to the forefront thanks to their effective use in vaccinations, vaccines and drug delivery, gene therapy, and diagnostic component transporters [8]. Virus-like particles (VLPs) are copycat to natural virions [13] and have been used as vehicles to carry foreign epitopes either by genetic fusion or by chemical conjugation [13] capable of inducing cellular and humoral responses [7, 13, 39]. Generally, this biological nanoparticle vaccine is safe, stable, highly immunogenic and protective, inexpensive to produce and cost-effective to supply. For example, porcine parvovirus (PPV) VLPs produced by self-assembly through in vivo or in vitro methods could effectively display a foreign epitope [14, 39] and are good particles for designing and developing chimeric epitope-based vaccines against PPV and FMDV [42]. Hence, similar to porcine parvovirus [28, 30] human parvovirus [2] and canine parvovirus [11], studies have indicated that BPV can be a key factor in the development of nanoparticle vaccines [18]. VLPS of human parvovirus and other parvoviruses are determined to be thermally and PH-stable. Similarly, the BPV virus has been predicted but not experimentally confirmed to have desirable properties for such platforms, including a high degree of acid and heat stability [17].

According to the findings of one study, BPV may be a useful tool to be used as a biologistics platform for deploying an epitope-based vaccine against FMDV [12]. Additionally, VLPs of BPV have been shown to stimulate an antiviral humoral and cellular immune response in animal models of BPV infection [42]. Similarly, the BPV virus has been shown to have desirable properties for such platforms, including a high degree of acid and heat stability [17]. Although BPV-VP2 assembles into VLPs in the absence of VP1, evidence suggests that VP1 may function in particle stability. Recent research suggests that VP2 or a combination of VP1 and VP2 can be used to generate virus-like particles (VLPs) via heterologous expression in eukaryotic cells, followed by in vitro self-assembly. [15]. However, the exact function of VP1 in BPV VLP formation remains unknown. The possibility of VLP synthesis by combining the VP1 and VP2 capsid proteins has not yet been demonstrated experimentally in a liquid laboratory. This study set out to answer the question, “How well might BPV virus-like particles (VLPs) be made from a single structural protein (VP2) and by combining the amino acid sequences of VP2 and VP1?.”

In this study, in silico-cloning of the capsid proteins, physicochemical characteristics and functional predictions of the immunodominant DE-loop were carried out bioinformatically before experimentation. The computational analysis served as good preliminary data for continuing the liquid experimentation.

Subsequently, an adequate amount of properly folded BPV capsid proteins was produced using the Bac-to-Bac Baculovirus Expression System as reported in [25]. Additionally, highly purified and homogeneous VLPs from sf9 cells were obtained by concentrating VLPs in 8% polyethylene glycol 8000 and 0.5 M NaCl and applying sucrose density gradient methods. Our findings show that VP2 alone was equally expressed and purified into detectable proteins, and the stability at different temperatures and pH values was not appreciably different between the two kinds of VLPs. Furthermore, BPV-VP2 VLPs were praised for their greater purity and integrity than BPV-VP1VP2 VLPs, as indicated by SDS‒PAGE. Therefore, our research demonstrates that the function of VP1 has no bearing on the stability or integrity of BPV-VLps. This is consistent with the notion that the VP2 protein is the primary subunit making up the BPV capsid.

Furthermore, the remarkable thermostability of BPV-VP2 VLPs was demonstrated by the fact that they maintained their structural integrity, but not their morphological size, after being incubated at temperatures ranging from 20 to 80 °C. This finding agrees with what was predicted by bioinformatics as detailed in the introduction and with the thermostability of human parvovirus B19 virus-like particles [32]. Our results are consistent with a prior work that found that parvovirus VLPs cultured at 80 °C for 30 minutes maintained their structural integrity under TEM [32]. This incredible physicochemical stability of bovine parvovirus is likely due to the strong association of viral structural polypeptides with the host cell (eukaryotic)-derived nuclear matrix during replication, as the virus relies heavily on host cell functional and structural factors for its replication [9, 27].

Additionally, the VP2 protein is thought to have strong immunogenicity [64] and to include a large number of antigen epitopes [11]. To be very clear, the purpose of our study was not to show the immunogenicity of the expressed capsid proteins but to assess the role of the minor capsid protein vp1 in maintaining the integrity of the BPV VLPs exposed to physicochemical conditions and their secondary structure. In light of these findings, however, we plan to continue employing VP2 VLPs in our future trials as a good carrier for multiepitope-based vaccine production against various cattle diseases and as a multivalent vaccine against enteric viruses.

Our findings show that the solid laboratory-predicted physicochemical properties of the VLP protein were identical to those of in vitro self-assembled VLPs in liquid form. The VLPs' (produced in our lab) thermal and pH stability suggests that they could be used for storage and formulation in the veterinary and medical fields, including production peptide vaccines and diagnostic kits. The anticipated grand average of hydropathicity (GRAVY) was negative, indicating that the protein was hydrophilic in nature and could interact with water molecules. The experimentally synthesized VLPS exhibits similar features to the predictive value. The VLPs of BPV are a good vehicle for the synthesis of vaccines against several infectious diseases, which may be due to the high degree of acid and heat stability [17]. Additionally, according to few reports that are currently available, the BPV structural surface protein can be used to recognize receptors and to choose a tissue or host, as well as to determine the pathogenicity and antigenicity of the virus [11]. More interestingly, our lab is the first to reverse-synthesize pAb of DE-loop in rabbit as an antigenic detection for different assays, including western blotting and IFAT, which previously needed a polyclonal antibody generated against the wild virus.

## Conclusion

In conclusion, we used both computational and experimental methods to generate recombinant BPV-pFastBac capsid proteins. We used insect expression platforms to describe and compare the abilities of recombinant BPV VP2 and VP1 VP2 cap proteins, as well as the role of VP1 in self-assembly into VLPs of the virus, and to the best of our knowledge, this is the first work for its kind. The BPV VP2-derived VLPs were found to be a promising candidate to carry heterogeneous epitopes in the development of a wide range of third-generation vaccinations and immunodiagnostic kits for various economically important animal diseases such as FMDV. These BPV-VLP vaccines could also be used to protect cattle from BPV infection. The insect cell system has been shown to be a flexible and potent technology for efficiently manufacturing VLPs in a short amount of time. Finally, the DE-loop Polyclonal antibody, which was antigenically predicted and manufactured, shows great promise for immunoassay detection and may also find application in diagnostics.

## Supplementary Information

**Additional file 1: Fig. S1.** Virus particles (natural virus) and Virus-like particles (VLPs) have comparable structures, are easily recognized by the immune system, and deliver viral antigens in a manner similar to the legitimate conformation, eliciting a robust immune response. **Fig. S2.** The BPV genome, depicted above in schematic form, is approximately 5.5 kilobases in size and is framed by a pair of nonidentical palindromic terminal hairpins. ORF1, ORF2, and ORF3 are the three largest open reading frames. In the genome (740–5307 kb) , ORF1 (740-2920 bp; left ORF) encodes a nonstructural protein (involved in DNA replication). ORF2 (middle ORF) encodes a nuclear phosphoprotein (2661-3302 bp) involved in viral RNA processing during gene expression. Two viral capsid proteins, VP1 and VP2, are encoded by ORF3 (right ORF) (3286-5307 bp) due to alternative splicing events. Together, VP1 and VP2 account for the bulk of the genome, approximately 2022 base pairs (bp), and VP2 is also responsible for the vast majority of capsid formation. There are flip inversion areas and hairpin loop stem loops at both the N and C termini (Agbandje-McKenna, 1998; Sukhu et al., 2013).

## Data Availability

Data will be made available on request.

## Abbreviations

CPE: Cytopathic effect
MOI: Multiplicity of infection
VP: Viral protein
VLPs: Virus like protein
BPV: Bovine parvovirus
CD: Circular dichroism
