# Correction: Stability and integrity of self-assembled bovine parvovirus virus‑like particles (BPV‑VLPs) of VP2 and combination of VP1VP2 assisted by baculovirus-insect cell expression: a potential logistical platform for vaccine deployment

**DOI:** 10.1186/s12985-026-03112-6

**Published:** 2026-04-11

**Authors:** Ashenafi Kiros Wubshet, Guo-xiu Li, Qian Li, Jun-Fei Dai, Yao-Zhong Ding, Luoyi Zhou, Min Qu, Yang Wang, Zhongyuan Ma, Gebremeskel Mamu Werid, Birhanu Hadush Abera, Asmelash Tassew Kebede, Yuefeng Sun, Xiangping Yin, Yongsheng Liu, Zhang Jie

**Affiliations:** 1 State Key Laboratory of Veterinary Etiological Biology, National/OIE Foot and Mouth Disease Reference Laboratory, Lanzhou Veterinary Research Institute, Chinese Academy of Agricultural Sciences, Lanzhou, 730046 People’s Republic of China; 2 College of Animal Science & Technology (CAST), Hebei Normal University of Science & Technology (HNUST), Qinhuangdao, People’s Republic of China; 3 Department of Veterinary Basics and Diagnostic Sciences, College of Veterinary Science, Mekelle University, 2084 Mekelle, Tigray Ethiopia; 4 Department of Animal Science, College of Agriculture and Natural Resources, Raya University, 92, Maychew, Tigray Ethiopia; 5 Davies Livestock Research Centre, School of Animal & Veterinary Sciences, University of Adelaide, Roseworthy Campus, Roseworthy, SA 5371 Australia

**Correction: Virology Journal (2024) 21:87**

10.1186/s12985-024-02322-0

In this article [1], Fig(s) 7, 10, 11 and 12 appeared incorrectly and have now been corrected in the original publication. For completeness and transparency, both correct and incorrect versions are displayed below.

The original article has been corrected.

Incorrect Fig. 7:



Corrected Fig. 7:

**Fig. 7:**
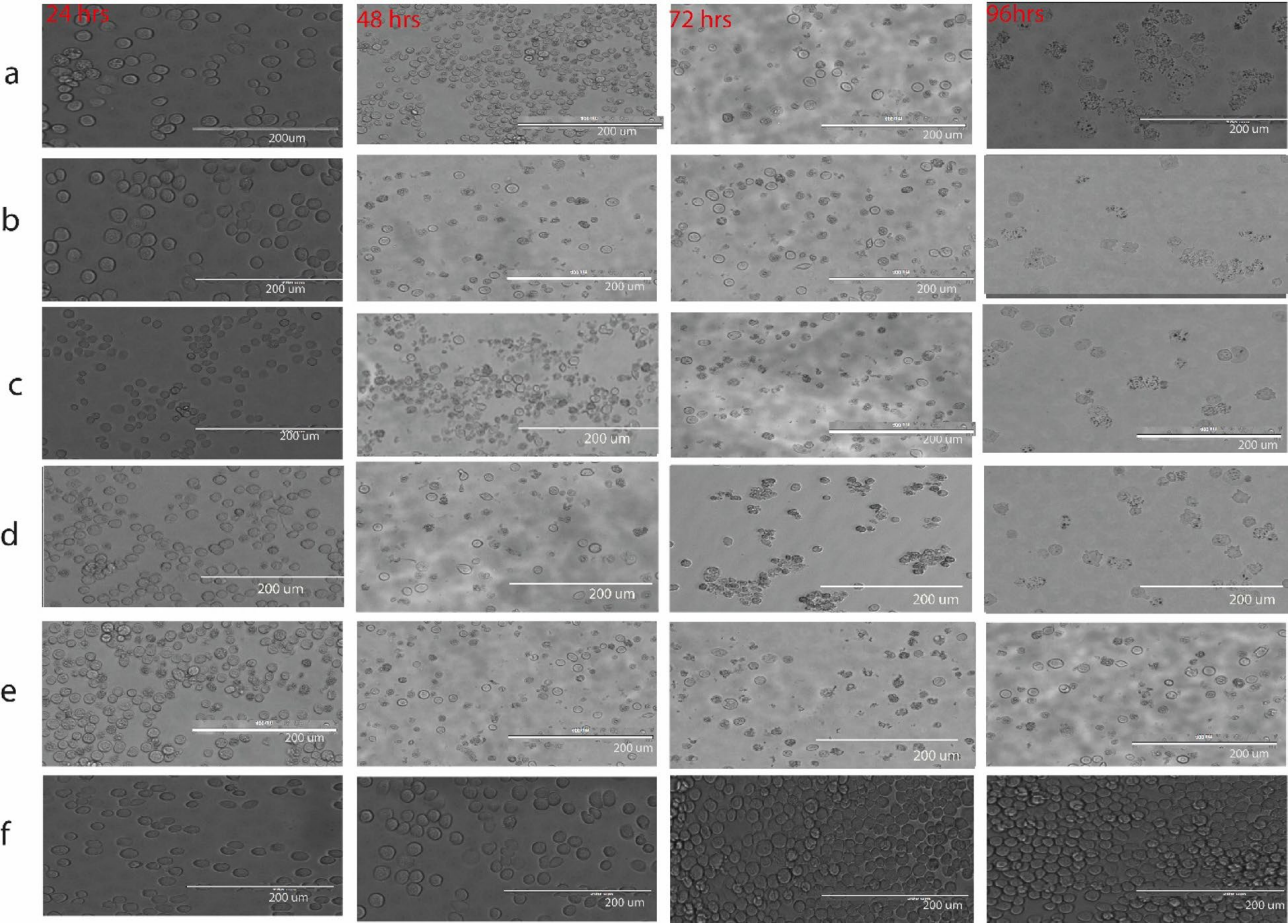
Four recombinant BPV-viral clones (VP1VP2 His-tagged, VP1VP2 without His-tag, VP2 His-tagged and VP2 without His-tag) were transfected into Sf9 cells using freshly recovered bacmids. For transfection, we employed cellfectin (2 ng Bacmid/8 ng cellfectin) to create recombinant baculovirus stocks (**a**–**d**). **a** VP2 His-tagged (BPV-VP2 with 6xhistag), **b** VP2 without His-tag (BPV-VP2 without 6xhistag), **c** VP1VP2 His-tagged (BPV-VP2VP1 with 6xhistag), **d** VP1VP2 without His-tag (BPV-VP2VP1 without 6xhistag), **e** PfastBacdual (positive control) **f** Mock (cell only). All recombinants showed an increase in cell width by 16.79 to 18. At a thickness of 79 µm 24 h after infection, 48 h pi cells were full of nuclei and no longer dividing, and 72- and 96-h pi cells showed a granular appearance (20.23 µm), detachment, and lysis. However, negative controls (cells only) appear spherical and somewhat granular (of normal size) during all phases of infection. 96 h in, it had a firm attachment on the surface, was vigorously dividing, and had become overly confluent

Incorrect Fig. 10:



 Corrected Fig. 10:

**Fig. 10:**
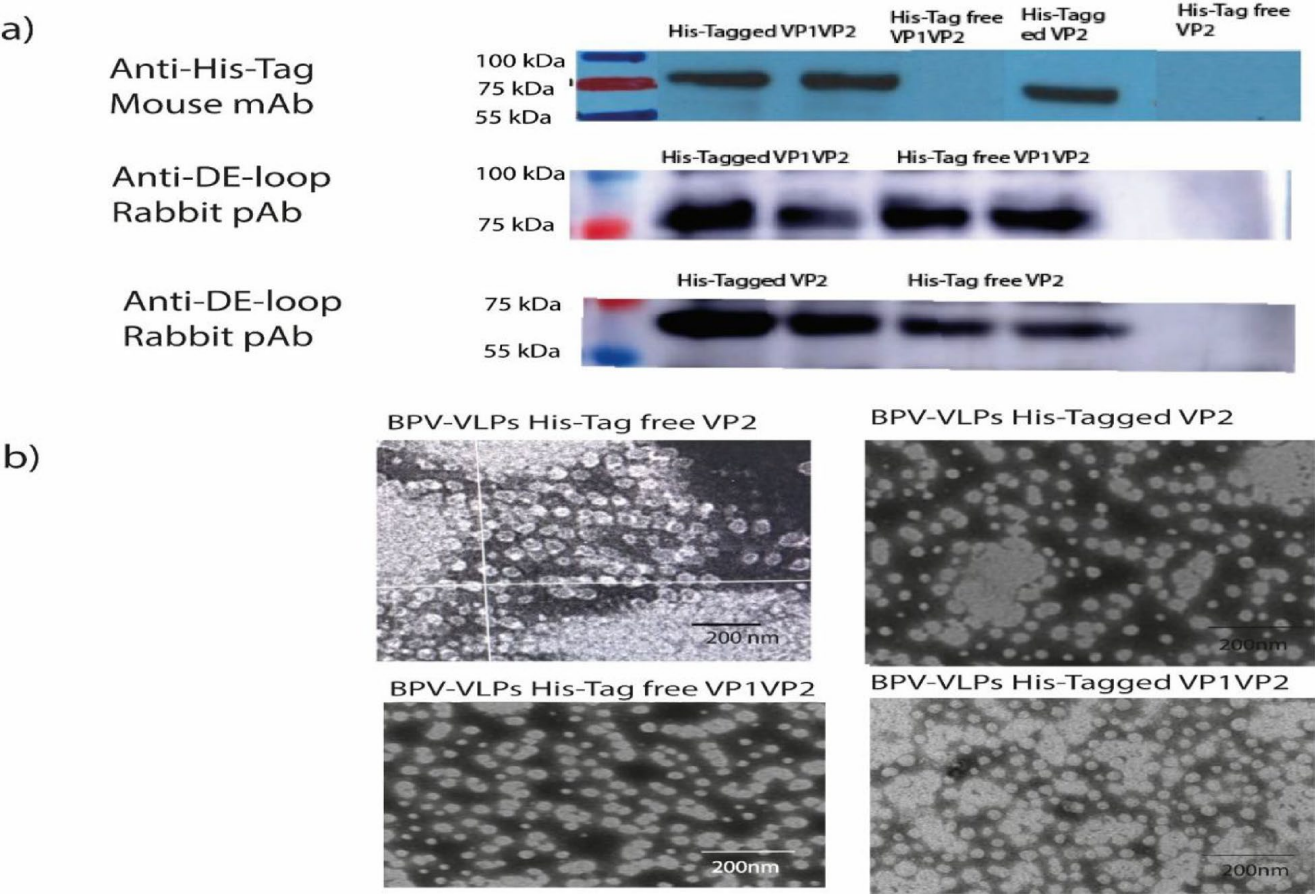
Western blot analysis of purified recombinant protein and negatively stained VLPs under a transmission electron microscope, **a** Purified VP1VP2 and VP2 recombinant proteins, with molecular weights of 75 kDa and 60 kDa, were recognized by an anti-His-tag monoclonal antibody and anti-DE-loop polyclonal antibody derived from BPV-VP2, **b** This TEM image demonstrates that BPV capsid proteins, both VP2 alone and VP1VP2, self-assembled to form ≈25 nm VLPs with uniform morphology

Incorrect Fig. 11:



Corrected Fig. 11:

**Fig. 11:**
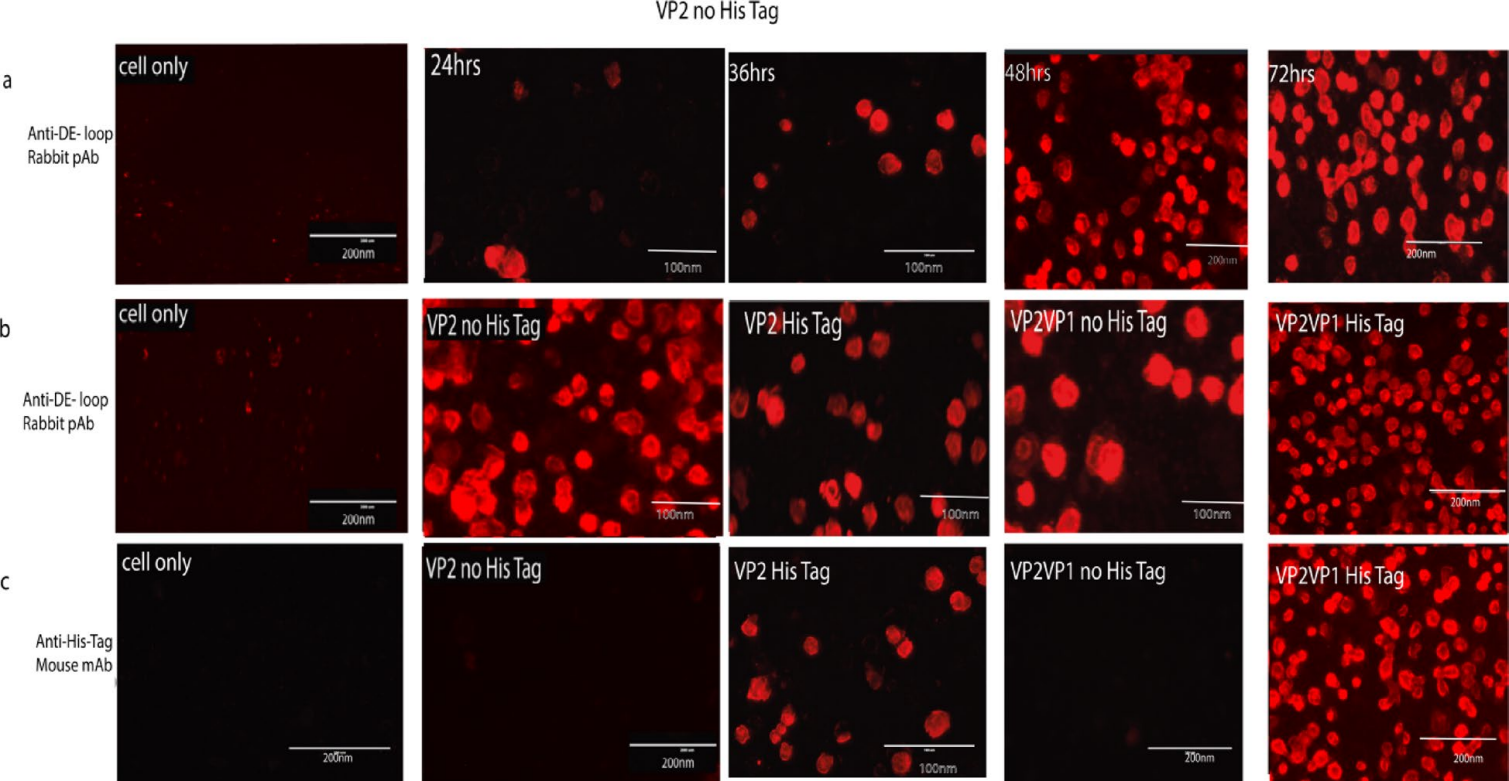
Illustrates the expression levels of recombinant proteins and their specific reactions with different antibodies, specifically VP1VP2 and VP2 alone, in Sf9 cells that were infected with recBacmid BPV: VP2 and recBacmid BPV: VP1VP2 at different time points. In all the panels the first in the left column represents the mock (cell only) as negative control, **a** At 24-, 48-, and 72-h post-transfection, the first panel displays the cells infected only with recBacmid BPVVP2 without His-tag, together with the level of expressed VP2 recombinant protein identified with pAb; **b** The second panel used anti-DE-loop rabbit polyclonal antibodies to identify His-tagged and His-Tag free VP1VP2 and VP2 protein expression levels; C The third panel displayed the expression levels of proteins from His-tagged and His-Tag free VP1VP2 and VP2 that were detected with anti-His-Tag mouse monoclonal antibodies. These proteins were His-tagged and His-Tag free. In this experiment, RFP (red fluorescence antibodies) Alexa-Fluor®-594-conjugated anti-rabbit IgG and antimouse secondary antibodies were used. Generally, the reaction in the fluorescence microscope clearly shows that the rec-proteins from different designs vary in expression at different time points, and the mAb and pAb are highly specific in the detection of the target proteins

Incorrect Fig. 12:



Corrected Fig. 12:

**Fig. 12:**
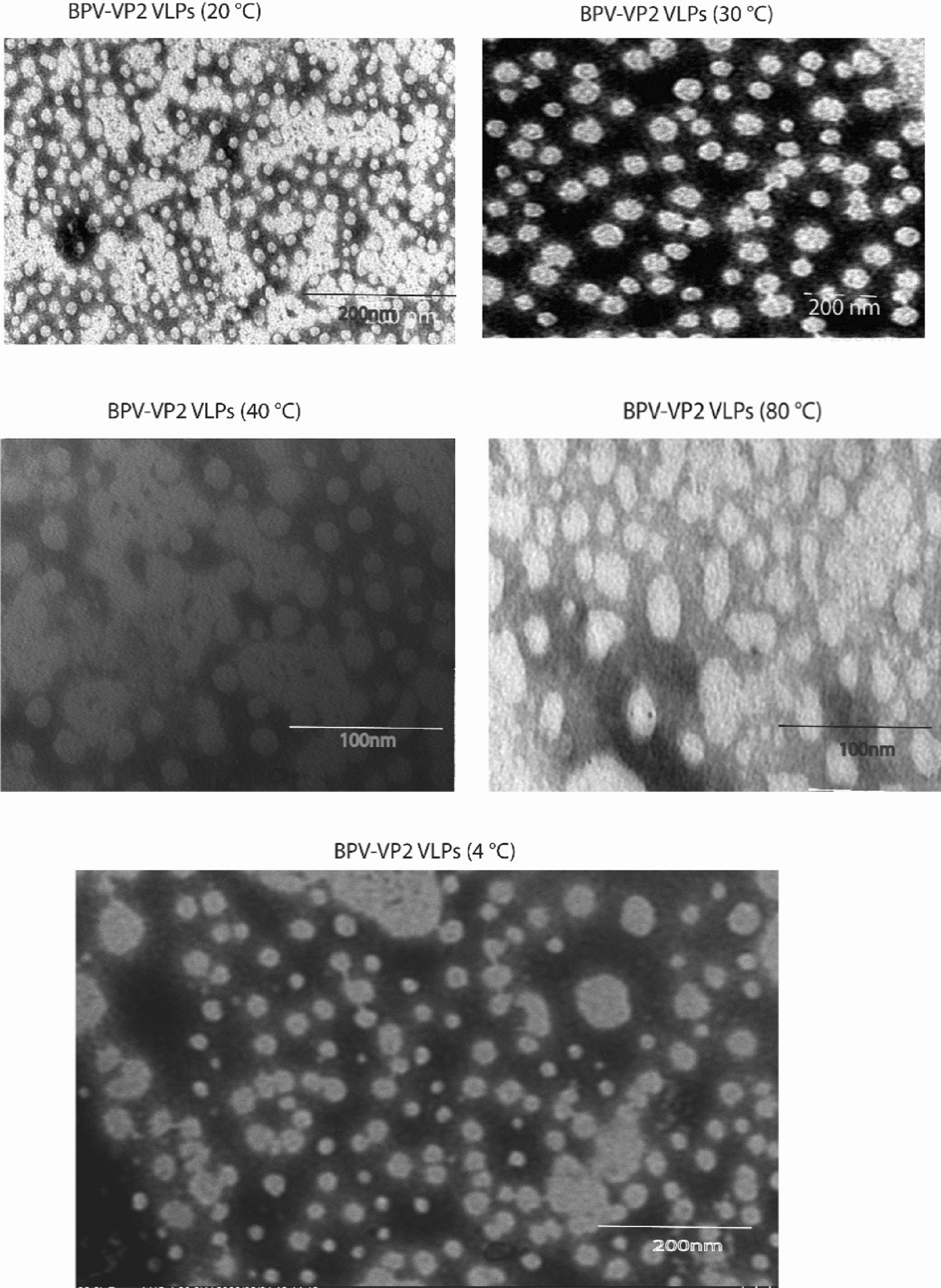
Transmission electron micrograph analyzing the morphological alterations and structural integrity of two types of VLPs following incubation at 20 °C, 30 °C, 40 °C, and 80 °C for 30 min to assess the thermal stability of BPV-VP2 VLPs in PBS

## References

[1] Wubshet AK, Li GX, Li Q, Dai JF, Ding YZ, Zhou L, Qu M, Wang Y, Ma Z, Werid GM, Abera BH, Kebede AT, Sun Y, Yin X, Liu Y, Jie Z. Stability and integrity of self-assembled bovine parvovirus virus-like particles (BPV-VLPs) of VP2 and combination of VP1VP2 assisted by baculovirus-insect cell expression: a potential logistical platform for vaccine deployment. Virol J. 2024;21:87. 10.1186/s12985-024-02322-0. 38641833 10.1186/s12985-024-02322-0 PMC11027344

