# Supplementary material for: Stability and integrity of self-assembled bovine parvovirus virus‑like particles (BPV‑VLPs) of VP2 and combination of VP1VP2 assisted by baculovirus-insect cell expression: a potential logistical platform for vaccine deployment
Source: Virol J. 2024 Apr 19;21:87. doi: 10.1186/s12985-024-02322-0 (PMC11027344; doi:10.1186/s12985-024-02322-0)
Supplement: Supplementary file 1 — Additional file 1: Fig. S1. Virus particles (natural virus) and Virus-like particles (VLPs) have comparable structures, are easily recognized by the immune system, and deliver viral antigens in a manner similar to the legitimate conformation, eliciting a robust immune response. Fig. S2. The BPV genome, depicted above in schematic form, is approximately 5.5 kilobases in size and is framed by a pair of nonidentical palindromic terminal hairpins. ORF1, ORF2, and ORF3 are the three largest open reading frames. In the genome (740–5307 kb) , ORF1 (740-2920 bp; left ORF) encodes a nonstructural protein (involved in DNA replication). ORF2 (middle ORF) encodes a nuclear phosphoprotein (2661-3302 bp) involved in viral RNA processing during gene expression. Two viral capsid proteins, VP1 and VP2, are encoded by ORF3 (right ORF) (3286-5307 bp) due to alternative splicing events. Together, VP1 and VP2 account for the bulk of the genome, approximately 2022 base pairs (bp), and VP2 is also responsible for the vast majority of capsid formation. There are flip inversion areas and hairpin loop stem loops at both the N and C termini (Agbandje-McKenna, 1998; Sukhu et al., 2013). [file 12985_2024_2322_MOESM1_ESM.zip › 12985_2024_2322_MOESM1_ESM/Supplementary table1. revised.docx]

Supplementary tables;

Table S1. Available strains, accession numbers and years of isolation of bovine parvovirus across different countries

| No | Acc.No | | Sequence | Host | Sample type | Isolate (strain) | Country of origin | Year | Reference |
| --- | --- | --- | --- | --- | --- | --- | --- | --- | --- |
| 1 | NC00150.1  M14363.1 1 | | Full genome | Bovine | Feces | Abinanti strain | USA | 1986 | (Chen, 1986). |
| 2 | NC038895 | Full genome | | Bovine | Feces | Abinanti strain | USA | 1988 | (Chen, 1988). |
| 3 | MW032437.1 | | Full genome | Yalk |  | Bovine parvovirus 1 | China | 2020 | (Shao et al., 2021). |
| 4 | JN191349 | | Partial VP1 VP2 | Bovine |  | Haden strain | China | 2011 | Unpublished |
| 5 | MN567095  MN567101 | | Partial VP2 | Bovine |  | Strain  VR-767 | Chin  a | 2016-  2017 | (Wang et al., 2019). |
| 6 | AF406967 | | Full genome | Bovine | Bovine sera | Bovine parvovirus 2 and 3 | USA |  | (Tobias Allander, 2001). |
| 7 | No reference number | | No information | Bovine |  | Proposed as HADEN 1 and 2 and C134 viruses | Japan | 1973 | (Inaba, 1973). |
| 8 | GenBank NP_954920. | | Full genome | Bovine |  | Bovine parvovirus 3 | Egypt | 2022 | (Nagy et al., 2022). |

| **Parvovirus**  **Species** | **VLPS type** | **Expression system/cells** | **Logistic service of VLPs** | | **References** |
| --- | --- | --- | --- | --- | --- |
|  |  |  | **FMDV type** | **Epitope** |  |
| Porcine Parvovirus | Adenovirus derived-PPV-VLPS | HEK-293A cells | O serotype (VP1) | B-cell (141–160)  T -cell (200–213) & (21–40) | (Xing-Xia, 2013). |
| Porcine Parvovirus | PPV-VLPS | Insect cell | O serotype  (VP1) | B-cell (141–160)  T -cell (200–213) & (21–40) | (Xuebao, 2010 V). |
| Porcine Parvovirus | PPV-VLPS | Insect cell | O serotype  (VP1) | T-cell epitope (aa21 to aa40) | (Chang et al., 2019). |
| Porcine parvovirus | PPV-VLPs | Insect cell | SAT2 serotype (VP1) | B-cell (141–160)  T -cell (21–40) | (Qian Li, 2021)Unpublished |
| Bovine parvovirus | BPV-VP2-VLPs | Insect cell expression | O serotype  (VP1) | Conserved neutralizing epitope 8E8 | (Chang et al., 2019). |

Table S 1. This table discusses the most common parvoviral virus-like particles (VLPs) as a logistical platform for vaccine deployment.
